# Supplementary material for: A plant NLR receptor employs ABA central regulator PP2C-SnRK2 to activate antiviral immunity
Source: Nat Commun. 2024 Apr 13;15:3205. doi: 10.1038/s41467-024-47364-8 (PMC11016096; doi:10.1038/s41467-024-47364-8)
Supplement: Supplementary file 1 — Supplementary Information [file 41467_2024_47364_MOESM1_ESM.pdf]

A plant NLR receptor employs ABA central regulator PP2C-SnRK2 to activate antiviral immunity

Shen Huang, Chunli Wang, Zixuan Ding, Yaqian Zhao, Jing Dai, Jia Li, Haining Huang, Tongkai Wang, Min Zhu, Mingfeng Feng, Yinghua Ji, Zhongkai Zhang and Xiaorong Tao*

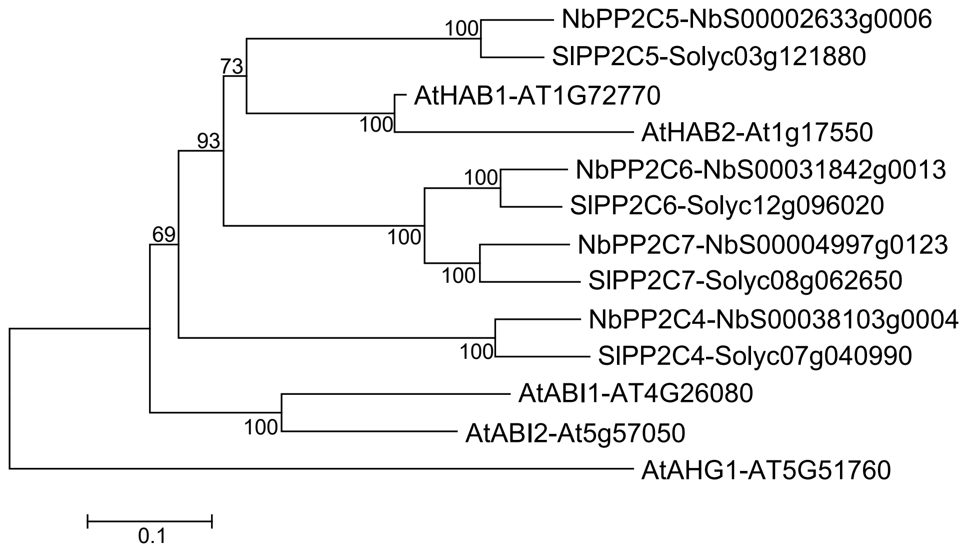

Supplementary Fig. 1 Phylogenetic analysis of group A PP2Cs from *Arabidopsis thaliana*, tomato, and *N. benthamiana* based on their amino acid sequences. Phylogenetic trees were constructed using MEGA 9 by the neighbor-joining method with 1000 bootstrap replicates. Detailed information for each group A PP2C with the protein accession number are included in the phylogenetic tree.

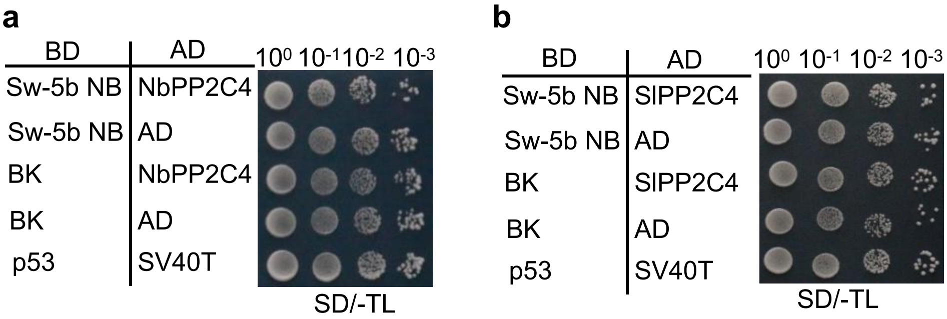

Supplementary Fig. 2 Y2H assay showing the direct interaction between Sw-5b NB and NbPP2C4 (a) or between Sw-5b NB and SlPP2C4 (b). The results for SD/-LT dropout media are shown in this Figure and results for SD/-LTHA dropout media are shown in Fig. 1a and 1h.

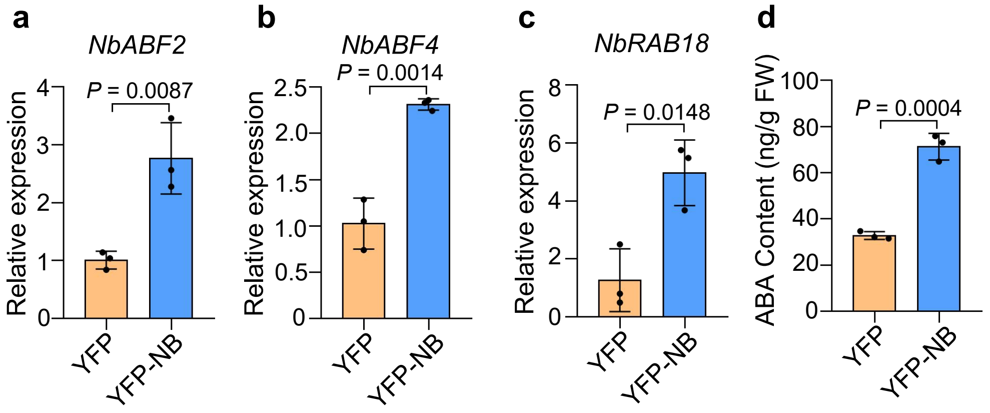

Supplementary Fig. 3 Transient overexpression of Sw-5b NB domain activates ABA responses in *N. benthamiana* leaves. (a–c) Relative expression levels of ABA-responsive genes *NbABF2* (a), *NbABF4* (b) and *NbRAB18* (c) in *N. benthamiana* leaves with transient overexpression of YFP-NB. Leaves transiently expressing YFP were used as controls. *NbActin* was used as the internal reference gene. Data are presented as means ± s.d. (n = 3 biologically independent samples). (d) The ABA content of *N. benthamiana* leaves with transient overexpression of YFP-NB. Leaves transiently expressing YFP was used as a control. Data were analysed by two-sided Student's t-test and shown as means ± s.d. (n = 3 biologically independent samples). *P* values are indicated in the graphs. Source data are provided as a Source Data file.

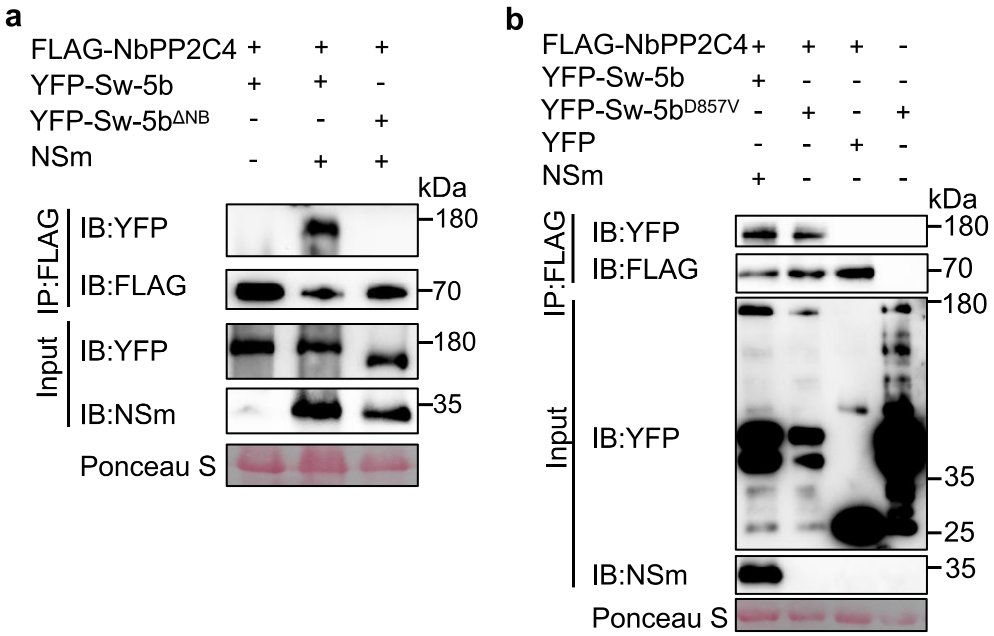

Supplementary Fig. 4 Co-immunoprecipitation assay (Co-IP) for the interaction between Sw-5b NB-deletion mutant (Sw-5b$^{\Delta NB}$) and NbPP2C4 or between Sw-5b^{D857V} auto-active mutant and NbPP2C4. (a) FLAG-NbPP2C4 was co-immunoprecipitated with YFP-Sw-5b or YFP-Sw-5b$^{\Delta NB}$ from leaf extracts of *N. benthamiana* with or without NSm. (b) FLAG-NbPP2C4 was co-immunoprecipitated with YFP-Sw-5b and NSm, YFP-Sw-5b^{D857V} or YFP control from leaf extracts of *N. benthamiana*. The blots were probed using YFP, FLAG or NSm specific antibodies. Ponceau S staining was used to estimate sample protein loadings. The protein size marker is shown on the right. All experiments were repeated at least three times with similar results.

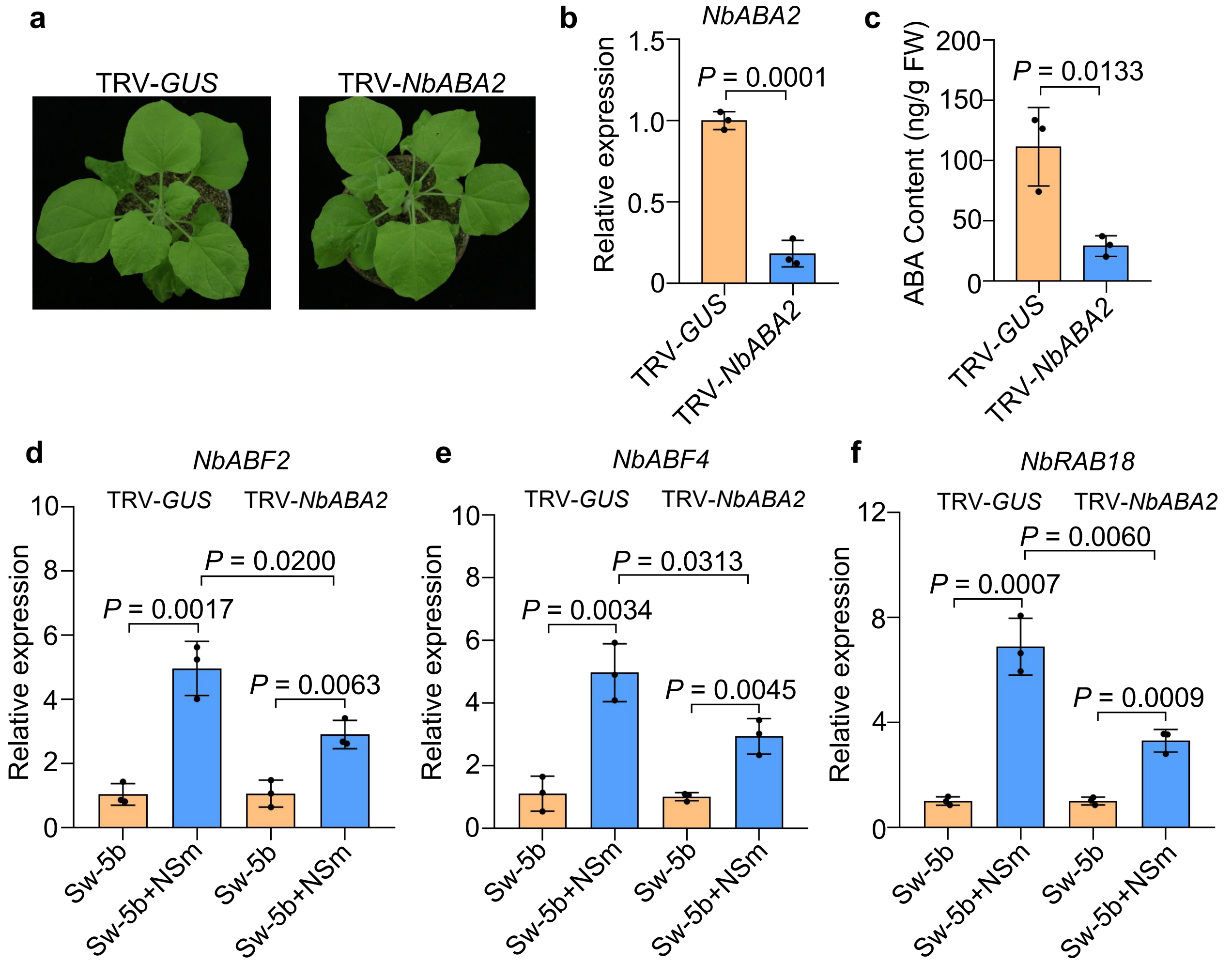

Supplementary Fig. 5 Sw-5b induces ABA signaling independent of ABA perception assayed in *N. benthamiana* plants. (a) Phenotypes of *NbABA2*-silenced or TRV-*GUS* control *N. benthamiana* plant. The mixture of *Agrobacterium* cultures harboring pTRV1 and pTRV2-*GUS* or pTRV1 and pTRV2-*NbABA2* were co-infiltrated into *N. benthamiana* plant leaves. Photographs were taken at 15 days post TRV treatment. (b) Relative expression levels of *NbABA2* in *N. benthamiana* plants treated with TRV-*GUS* or TRV2-*NbABA2* analyzed by qRT-PCR. Data are presented as means ± s.d. (n = 3). (c) Amount of ABA in *NbABA2*-silenced or TRV-*GUS* control *N. benthamiana* plants. Data are presented as means ± s.d. (n = 3). (d-f) The expression levels of ABA response genes *NbABF2*, *NbABF4* and *NbRAB18* in NbABA2-silenced or TRV-*GUS* control plants (co)expressing Sw-5b or Sw-5b and NSm.Data are presented as means ± s.d. (n = 3). Data were analysed by two-sided Student's t-test. *P* values are indicated in the graphs. Source data are provided as a Source Data file.

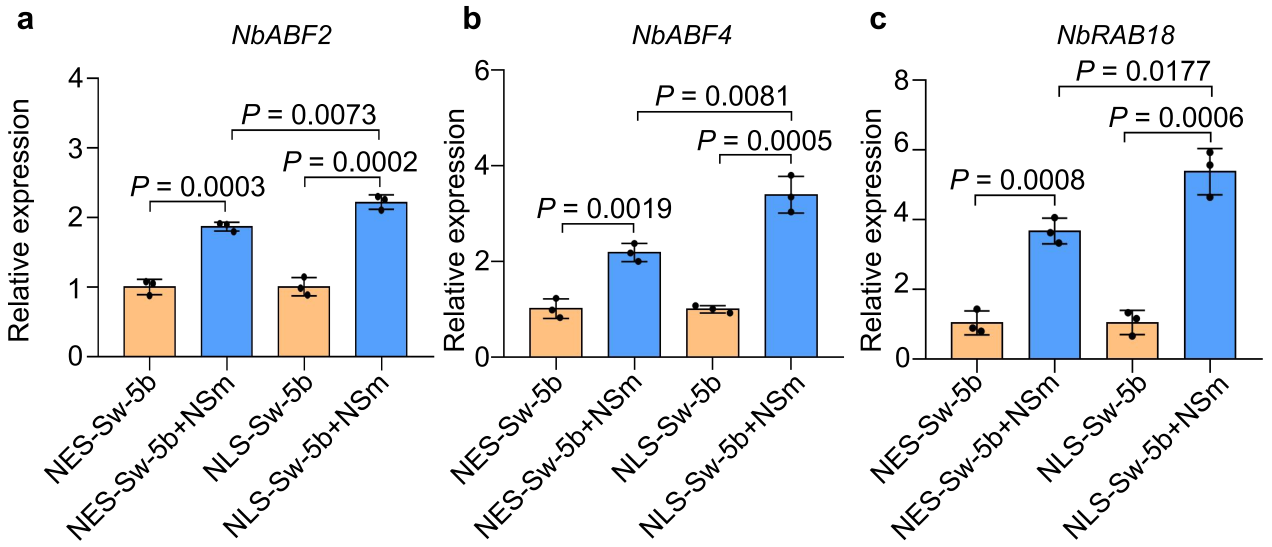

Supplementary Fig. 6 Both the cytoplasmic and nuclear pools of the Sw-5b NLR induce ABA signaling. (a-c) Relative expression levels of representative ABA responsive genes *NbABF2*, *NbABF4*, and *NbRAB18* in *N. benthamiana* leaves by (co)expressing NES-Sw-5b, NES-Sw-5b+NSm or NLS-Sw-5b, and NLS-Sw-5b+NSm. Nb*Actin* was used as the internal reference gene. *P* values are indicated in the graphs. Data are mean ± s.d. n = 3 biologically independent samples.

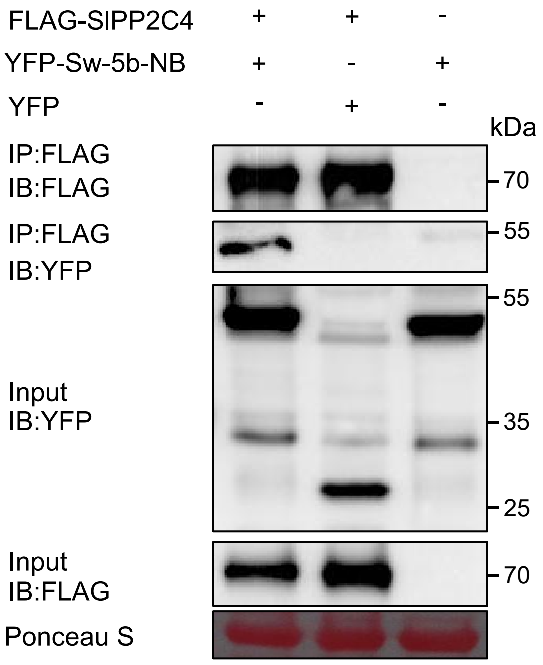

Supplementary Fig. 7 Co-immunoprecipitation assay (Co-IP) for the interaction between Sw-5b NB and SlPP2C4. FLAG-SlPP2C4 was co-immunoprecipitated with YFP-NB or YFP control from leaf extracts of *N. benthamiana*. The blots were probed using FLAG or YFP specific antibodies. Ponceau S staining was used to estimate sample protein loadings. The protein size marker is shown on the right. Experiments were repeated at least three times with similar results.

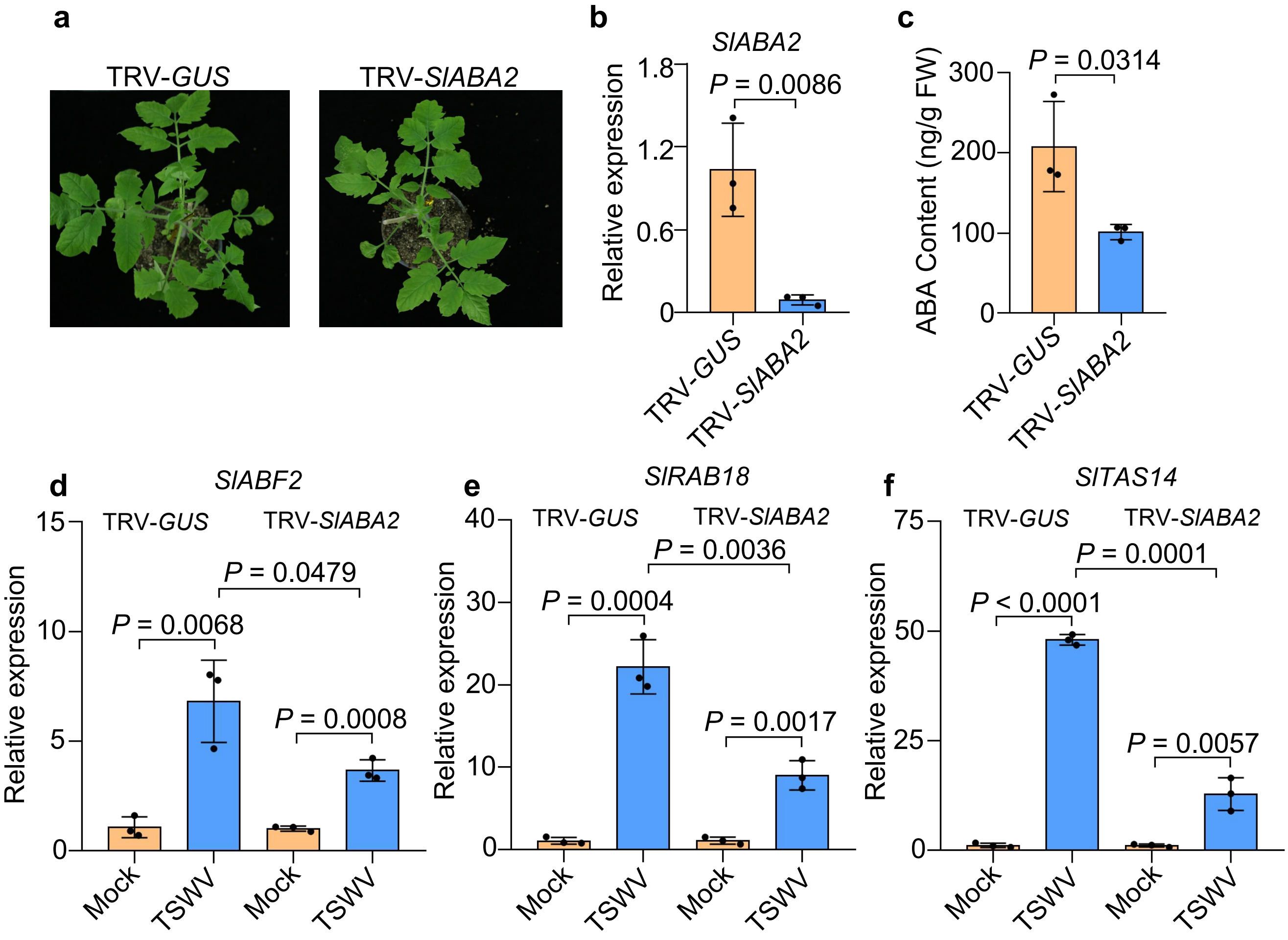

Supplementary Fig. 8 Sw-5b induces ABA signaling independent of ABA perception assayed in tomato plants. (a) Phenotypes of *SlABA2*-silenced or TRV-GUS control tomato plant. The mixture of *Agrobacterium* cultures harboring pTRV1 and pTRV2-*GUS* or pTRV1 and pTRV2-*SlABA2* were co-infiltrated into tomato plant leaves. Photographs were taken at 20 days post TRV treatment. (b) Relative expression levels of SlABA2 in tomato plants treated with TRV-*GUS* or TRV2-*SlABA2* analyzed by qRT-PCR. Data are presented as means ± s.d. (n = 3 biologically independent samples). (c) Amount of ABA in *SlABA2*-silenced or TRV-*GUS* control tomato plants. Data are presented as means ± s.d. (n = 3). (d-f) The expression levels of ABA response genes *SlABF2*, *SlRAB18* and *SlTAS14* in *SlABA2*-silenced or TRV-*GUS* control plants inoculated with TSWV at 4 dpi. *SlActin* was used as the internal reference gene. Data are presented as means ± s.d. (n = 3). Plants inoculated with phosphate buffer (PB) were used as a Mock control. Data were analysed by two-sided Student's t-test. *P* values are indicated in the graphs or Source data. Source data are provided as a Source Data file.

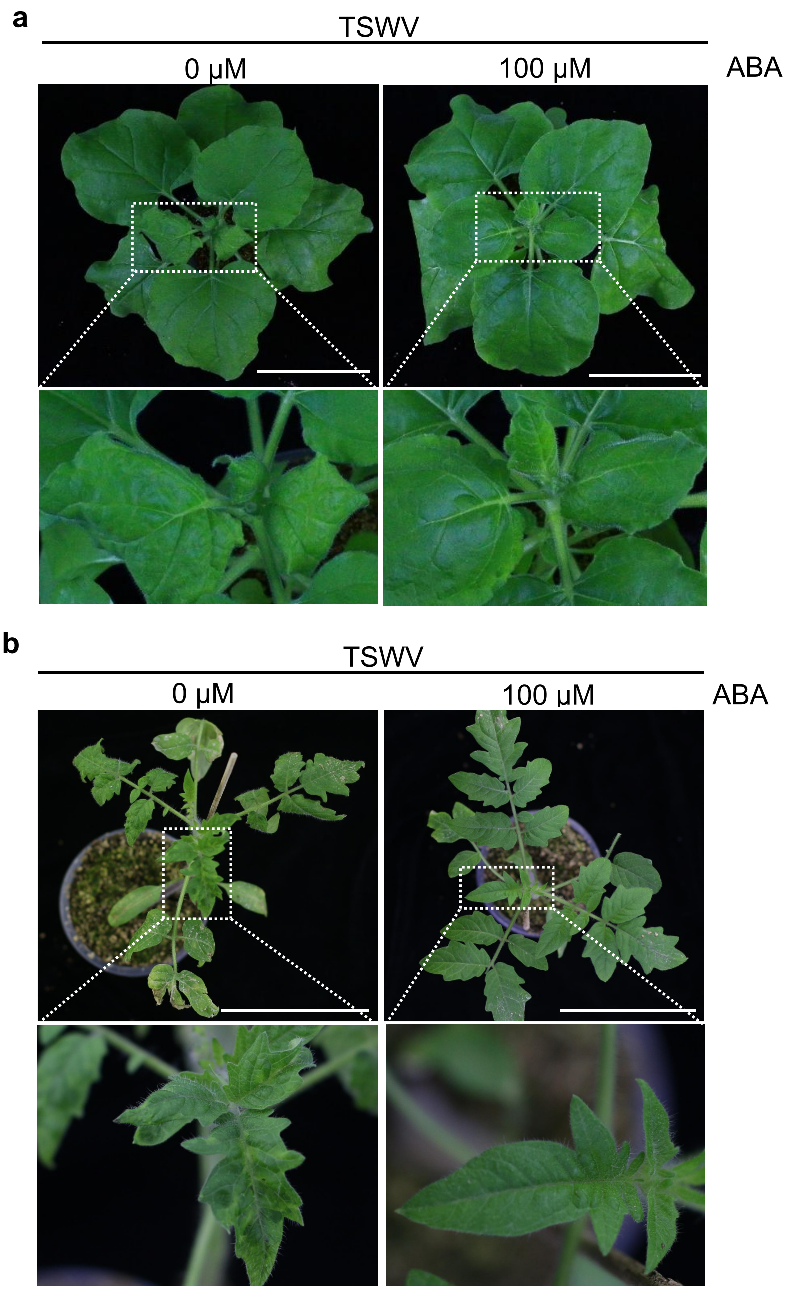

Supplementary Fig. 9 Phenotypes of TSWV-inoculated *N. benthamiana* and tomato plants treated with or without 100 μM ABA. At 24 h post treatment, the fresh sap from TSWV-infected leaf tissues was mechanically inoculated onto ABA-treated or control leaves. The phenotypes of TSWV-inoculated plants treated with or without ABA were photographed at 7 dpi.

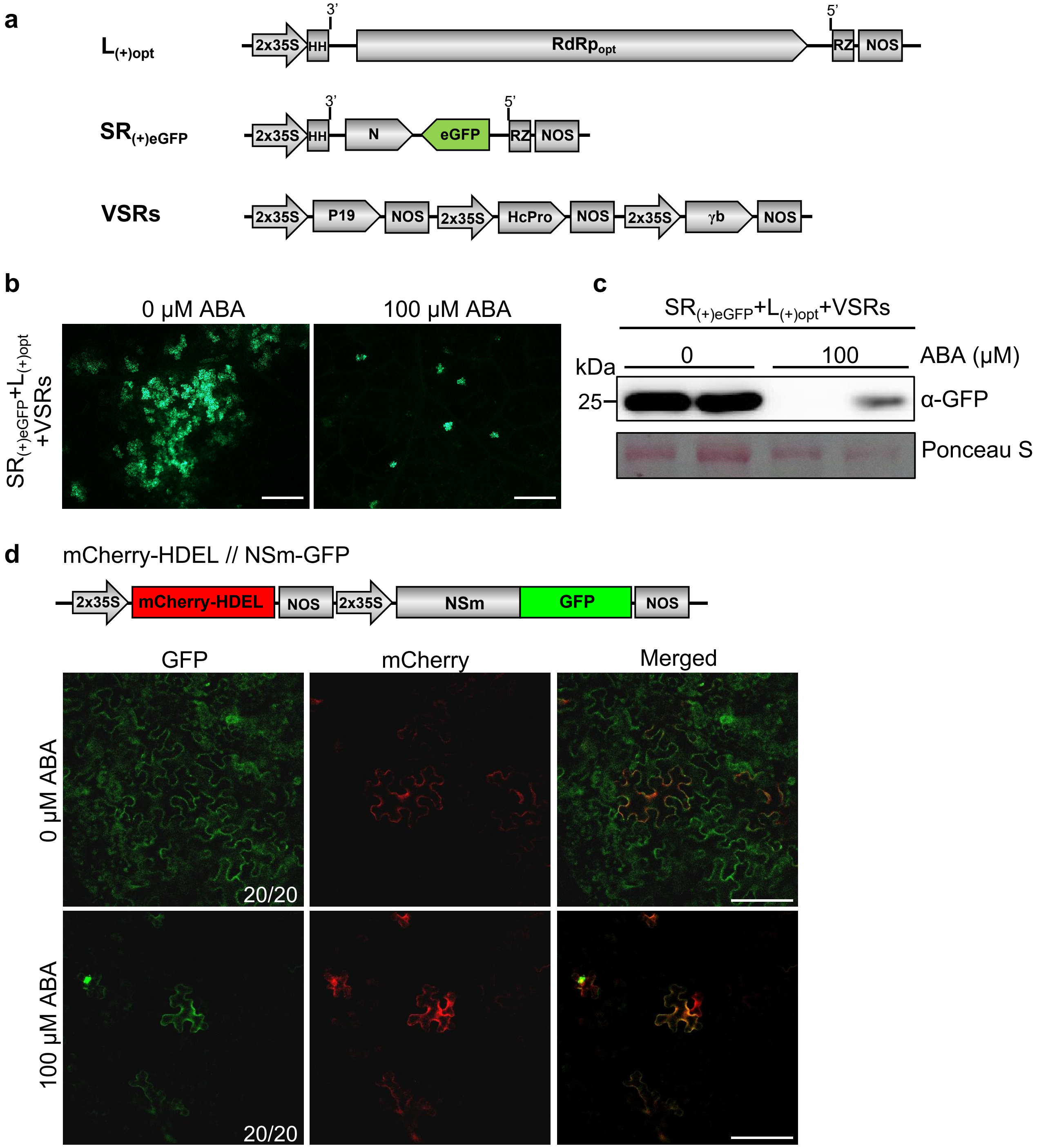

Supplementary Fig. 10 ABA treatment inhibits both viral replication and intercellular movement of TSWV. (a) Schematic representation of infectious clones of TSWV $L_{(+)opt}$ expressing RdRp, $SR_{(+)eGFP}$ minireplicon, and VSRs. $RdRp_{opt}$ represents optimized RdRp; VSRs, 3 RNA silencing suppressors P19, HcPro, and γb. (b, c) *N. benthamiana* plant leaves were treated with or without 100 μM ABA, followed by agroinoculation of TSWV $L_{(+)opt}$ + $SR_{(+)eGFP}$ + VSRs. The accumulation of

TSWV $SR_{(+)eGFP}$ minireplicon was examined at 3 dpi using inverted fluorescence microscopy or by western blot probed with GFP specific antibodies. Scale bars = 600 μm. (d) Cell-to-cell movement analysis of NSm-GFP in *N. benthamiana* leaves with or without ABA treatment. Schematic diagram of the mCherry-HDEL//NSm-GFP construct that co-express mCherry-HDEL and NSm-GFP from the same binary vector is shown in the upper panel. *Agrobacterium* containing the mCherry-HDEL//NSm-GFP construct was diluted 500 times for expression in a single epidermal cell. The cell-to-cell movement of NSm-GFP were analyzed under confocal microscopy at 28 h post agroinfiltration (lower panel). Scale bars = 100 μm. All experiments were repeated at least three times with similar results.

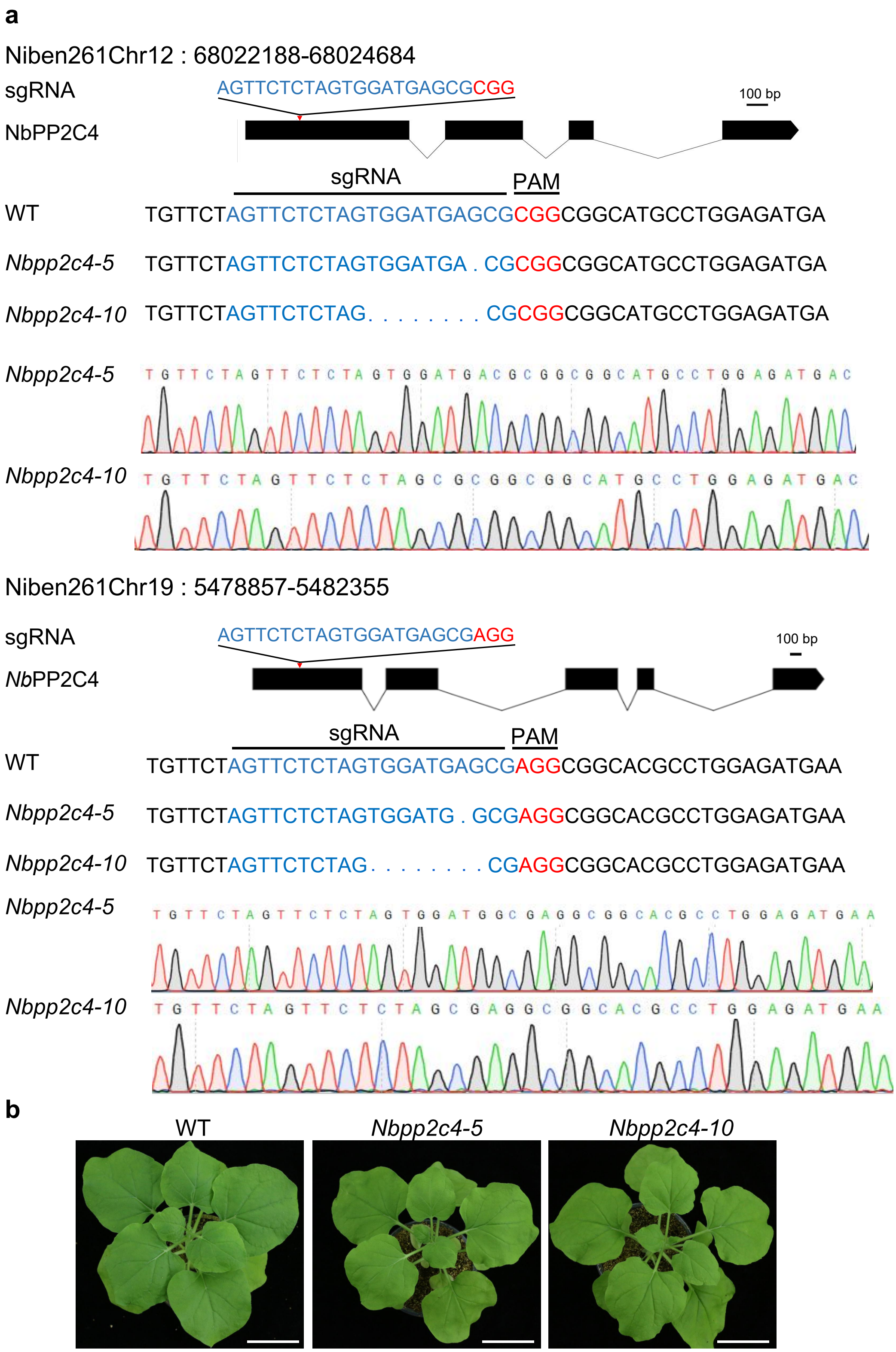

a
Niben261Chr12 : 68022188-68024684
sgRNA
AGTTCTCTAGTGGATGAGCGCGG
100 bp
NbPP2C4
sgRNA
PAM
WT
TGTTCTAGTTCTCTAGTGGATGAGCGCGGCGGCATGCCTGGAGATGA
Nbpp2c4-5
TGTTCTAGTTCTCTAGTGGATGA . CGCGGCGGCATGCCTGGAGATGA
Nbpp2c4-10
TGTTCTAGTTCTCTAG. . . . . CGCGGCGGCATGCCTGGAGATGA
Nbpp2c4-5
Nbpp2c4-10
Niben261Chr19 : 5478857-5482355
sgRNA
AGTTCTCTAGTGGATGAGCGAGG
100 bp
NbPP2C4
sgRNA
PAM
WT
TGTTCTAGTTCTCTAGTGGATGAGCGAGGCGGCACGCCTGGAGATGAA
Nbpp2c4-5
TGTTCTAGTTCTCTAGTGGATG . GCGAGGCGGCACGCCTGGAGATGAA
Nbpp2c4-10
TGTTCTAGTTCTCTAG. . . . . CGAGGCGGCACGCCTGGAGATGAA
Nbpp2c4-5
Nbpp2c4-10
b
WT
Nbpp2c4-5
Nbpp2c4-10

Supplementary Fig. 11 Generation of *Nbpp2c4* knockout *Nicotiana benthamiana* mutant lines using a CRISPR-Cas9 based gene editing technology. (a) Schematic diagram showing the genome structure of *NbPP2C4* and the location and sequence of the sgRNA. The black boxes represent the exons and the black lines represent the introns. Arrows indicate the position of sgRNA; the PAM motif (NGG) is shown in red. Sequence alignment and sequence chromatogram showing the gene editing sites in *Nbpp2c4-5* and *Nbpp2c4-10* mutant lines compared to the wild type (WT) *NbPP2C4* sequence. (b) A photograph showing 10-week-old WT *N. benthamiana* plant, *Nbpp2c4-5*, and *Nbpp2c4-10* knockout mutant lines.

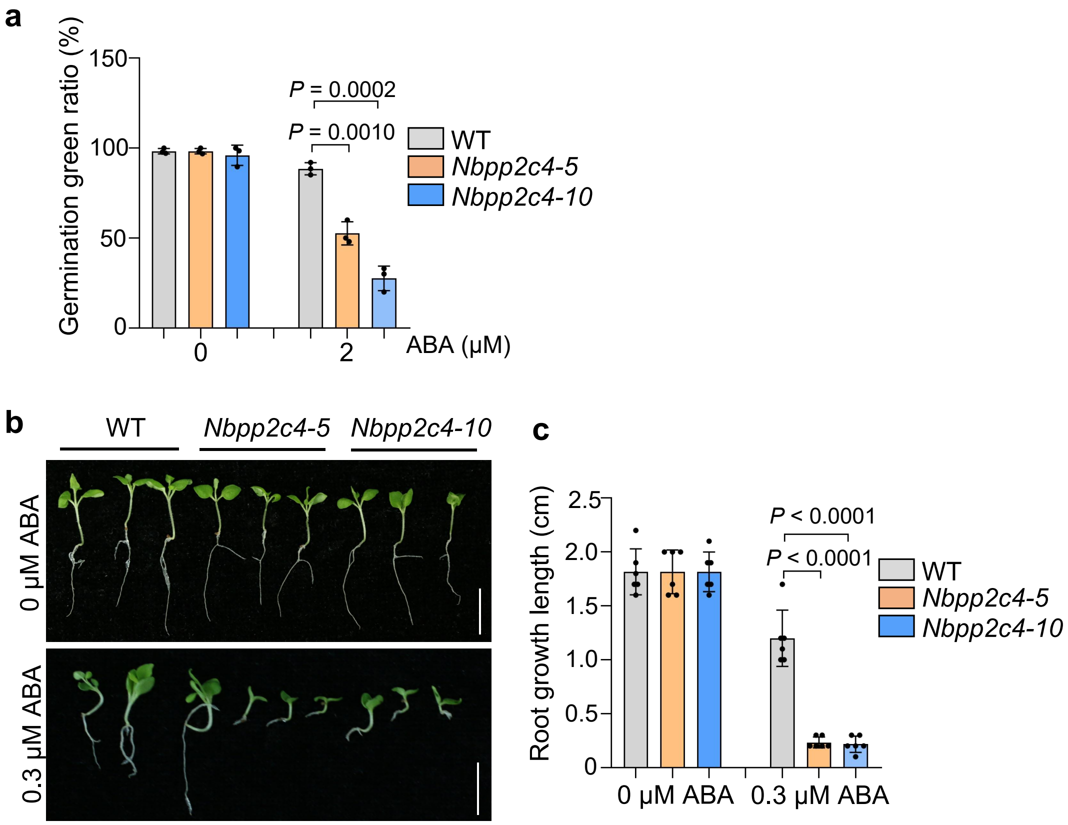

Supplementary Fig. 12 Seedlings of *Nbpp2c4* knockout mutants are hypersensitive to ABA treatment. (a) Seed germination analysis of the wild-type (WT) *N. benthamina* or *Nbpp2c4-5* and *Nbpp2c4-10* mutant seeds on MS medium supplemented with or without 2 μM ABA. Error bars represent ± s.d. (n = 3). (b) Primary root growth analysis of WT *N. benthamina* seedlings or *Nbpp2c4-5* and *Nbpp2c4-10* mutant seedlings vertically grown on MS medium supplemented with or without 0.3 μM ABA. Bars = 1 cm. The photograph was taken at 15 days post ABA treatment. (c) Statistic analyses of primary root length of wild-type (WT) and mutant plants treated with 0 or 0.3 μM ABA. Error bars represent ± s.d. (n = 6 plants). Data were analysed by two-sided Student's t-test. The exact *P* values are shown in the graphs or Source data. Source data are provided as a Source Data file.

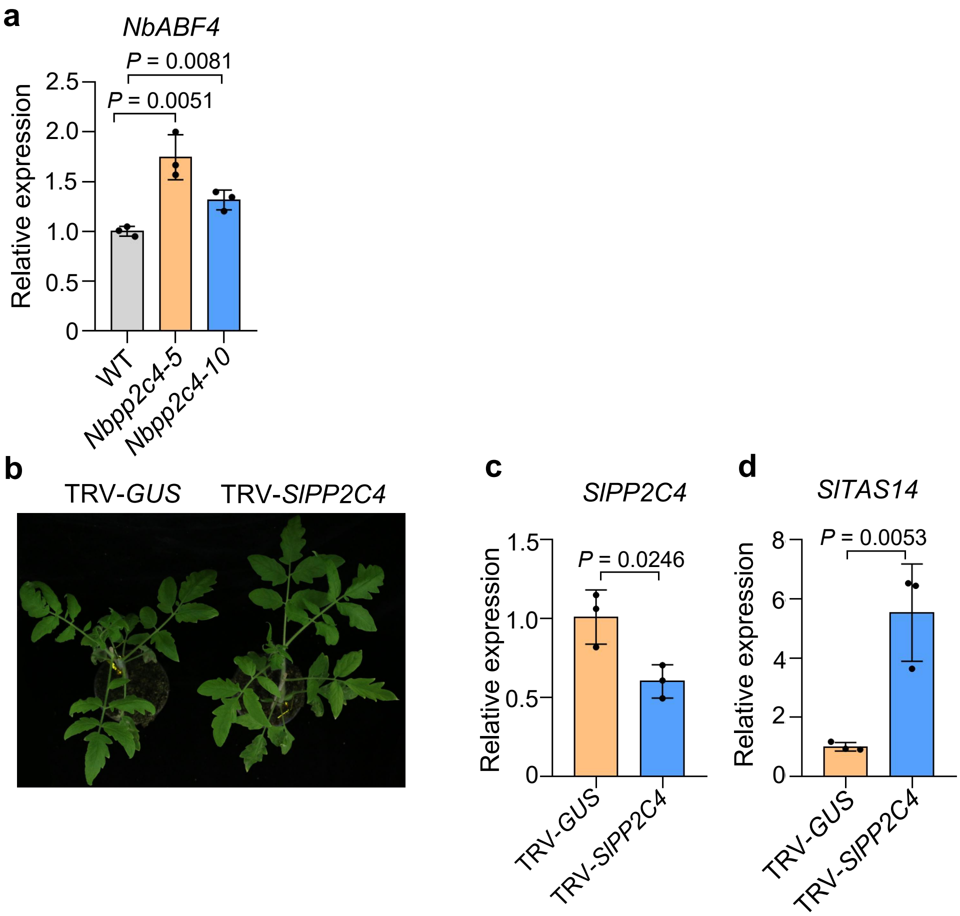

Supplementary Fig. 13 NbPP2C4/SlPP2C4 is a negative regulator of ABA signaling. (a) The expression levels of ABA response genes *NbABF4* in *Nbpp2c4-5* and *Nbpp2c4-10* mutants were significantly higher than those in wide-type (WT) *N. benthamiana* plants with the treatment of 100 μM ABA. *NbActin* was used as the internal reference gene. Data are presented as means ± s.d. (n = 3 biologically independent samples). (b) Phenotypes of *SlPP2C4*-silenced or TRV-*GUS* control tomato plant. The mixture of *Agrobacterium* cultures harboring pTRV1 and pTRV2-*GUS* or pTRV1 and pTRV2-*SlPP2C4* were co-infiltrated into tomato plant leaves. Photographs were taken at 30 days post TRV treatment. (c) Relative expression levels of *SlPP2C4* in tomato plants treated with TRV-*GUS* or TRV2-*SlPP2C4* analyzed by qRT-PCR. Data are presented as means ± s.d. (n = 3). (d) The expression levels of ABA response genes *SlTAS14* in *SlPP2C4*-silenced tomato plants were significantly higher than those in TRV-*GUS* control plants with 100 μM ABA treatment. Data are presented as means ± s.d. (n = 3). *SlActin* was used as the internal reference gene. Data were analysed by two-sided Student's t-test. *P* values are indicated in the graphs. Source data are provided as a Source Data file.

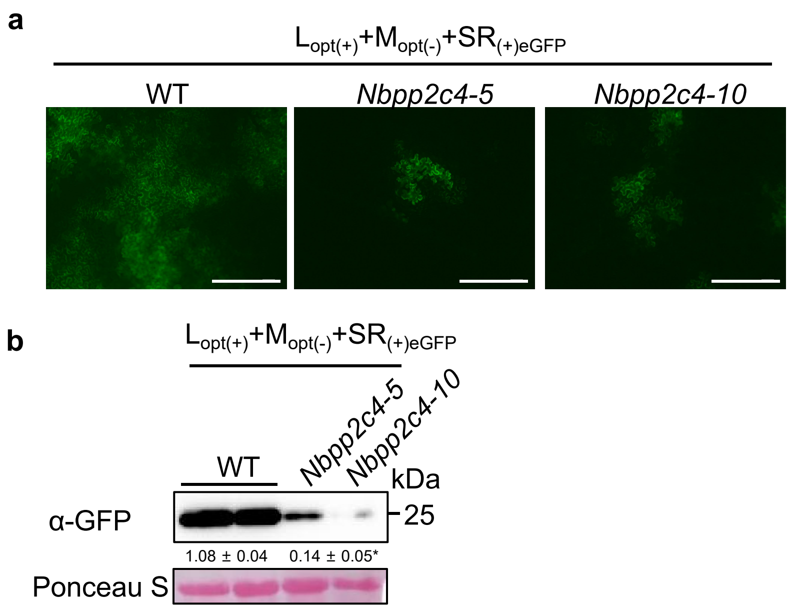

Supplementary Fig. 14 Knockout of *NbPP2C4* activates host plant defence against TSWV infection. (a) Wild-type (WT) *N. benthamiana*, *Nbpp2c4-4* or *Nbpp2c4-10* plants were inoculated with TSWV infectious clones $L_{(+)opt}$, $M_{(-)opt}$ and $SR_{(+)eGFP}$ carrying eGFP reporter via agro-infiltration. The eGFP fluorescence expressed from TSWV replicon in the agro-infiltrated *N. benthamiana* plant leaves were examined and photographed using an inverted fluorescence microscope at 2.5 days post agro-infiltration (dpi). Scale bars = 800 μm. (b) Immunoblot analysis of TSWV accumulation in the leaves shown in panel a using a GFP specific antibody. *, $P < 0.05$. All experiments were repeated at least three times with similar results.

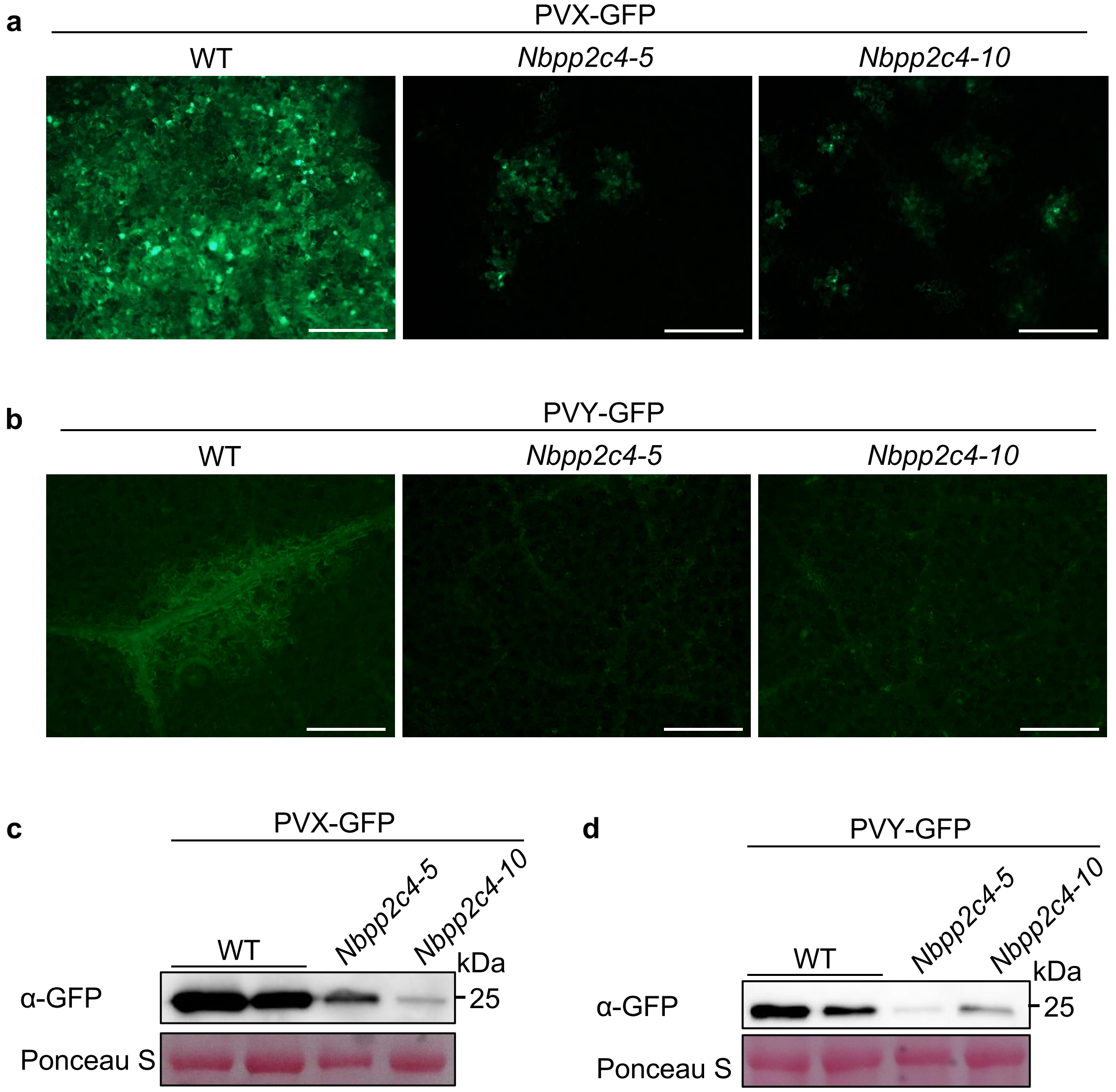

Supplementary Fig. 15 Knockout of *NbPP2C4* activates host plant defence against PVX and PVY infection. (a and b) Wild-type (WT) *N. benthamiana*, *Nbpp2c4-4* or *Nbpp2c4-10* plants were inoculated with an infectious clone of PVX-GFP (a) or PVY-GFP (b) via agro-infiltration. The eGFP fluorescence expressed from PVX-GFP or PVY-GFP in *N. benthamiana* plant leaves were analyzed and photographed using an inverted fluorescence microscope at 2.5 days post agro-infiltration (dpi). Scale bars = 300 μm. (c and d) Immunoblot analysis of PVX-GFP (c) or PVY-GFP (d) in WT, *Nbpp2c4-4* or *Nbpp2c4-10* using a GFP specific antibody. All experiments were repeated at least three times with similar results.

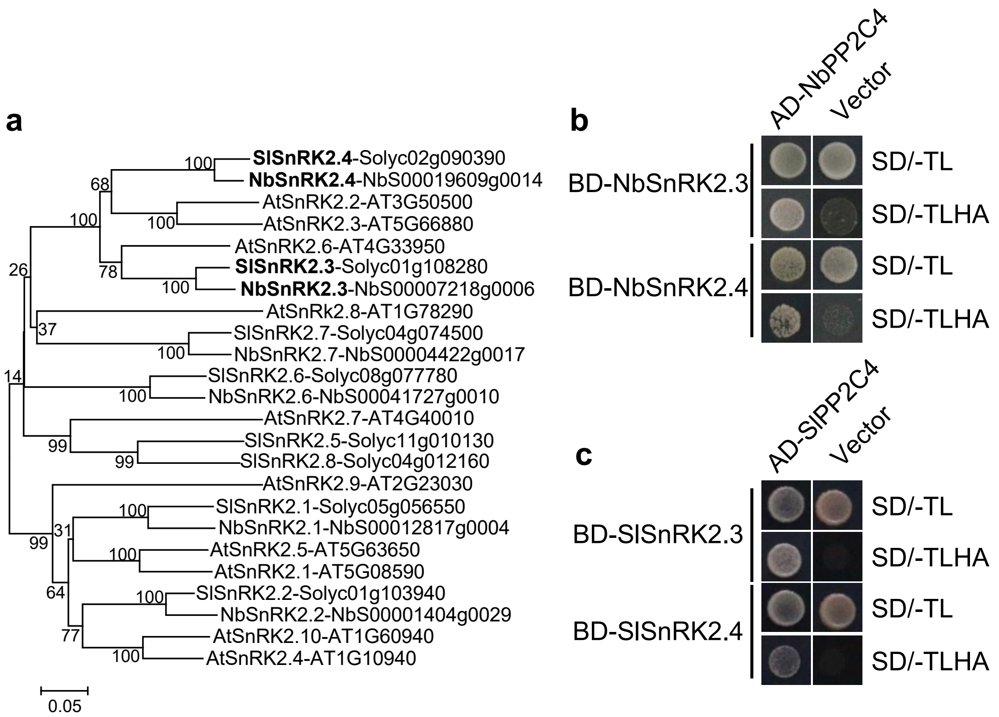

Supplementary Fig. 16 NbPP2C4/SlPP2C4 interacts with NbSnRK2.3/2.4 or SnRK2.3/2.4 in yeast. (a) Phylogenetic analysis of SnRK2 from *Arabidopsis thaliana*, tomato, and *Nicotiana benthamiana* based on their amino acid sequences. Phylogenetic trees were constructed using MEGA 9 by the neighbor-joining method with 1000 bootstrap replicates. Detailed information for each SnRK2 with the sequences accession number are included in the phylogenetic tree. (b and c) Y2H assay show the interaction between NbSnRK2.3/2.4 and NbPP2C4 (b) or the interaction between SlSnRK2.3/2.4 and SlPP2C4 (c). The BD-NbSnRK2.3/2.4 or BD-SlSnRK2.3/2.4 was co-transformed with AD-PP2C4 or AD control vector into yeast and their growth and interactions were assayed on SD/-LT and SD/-LTHA dropout media, respectively.

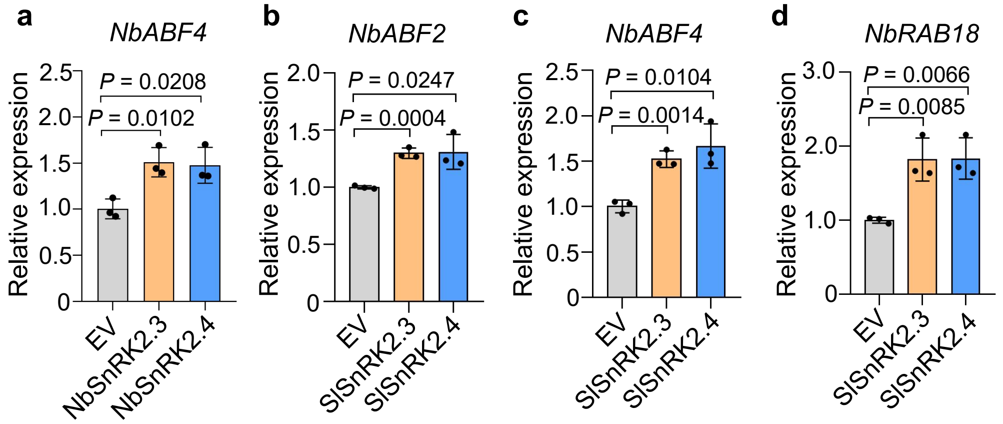

Supplementary Fig. 17 Overexpression of *NbSnRK2.3/2.4* or *SlSnRK2.3/2.4* upregulates ABA response genes in *N. benthamiana* plant. (a) Relative expression levels of ABA-response gene *NbABF4* in *N. benthamiana* plant leaves overexpressing *NbSnRK2.3* and *NbSnRK2.4* at 24 hours post agro-infiltration (hpai). The *N. benthamiana* plant leaves expressing p2300S empty vector (EV) control was used as a control. (b–d) Relative expression levels of ABA-response genes *NbABF2* (b), *NbABF4* (c) and *NbRAB18* (d) in *N. benthamiana* plant leaves overexpressing *SlSnRK2.3* or *SlSnRK2.4* at 24 hpai. *NbActin* was used as the internal reference gene. Data are presented as means ± s.d. (n = 3). Data were analysed by two-sided Student's t-test. *P* values are indicated in the graphs. Source data are provided as a Source Data file.

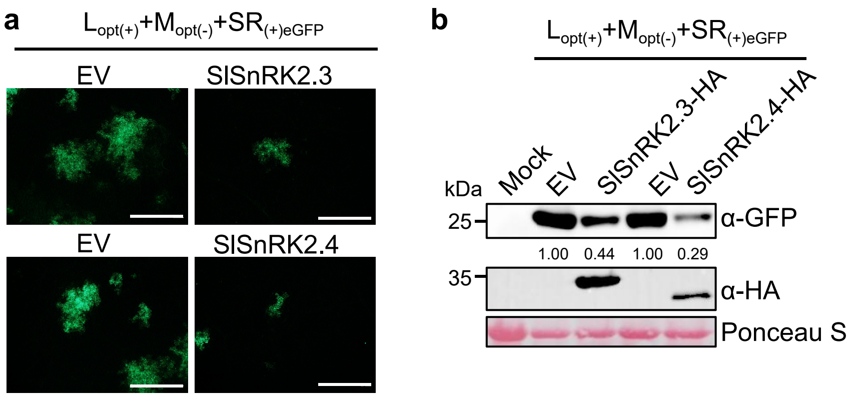

Supplementary Fig. 18 Transient overexpression of *SlSnRK2.3/2.4* induces host plant defence against TSWV infection in *N. benthamiana*. (a) Overexpression of *SlSnRK2.3/2.4* inhibits the accumulation of TSWV replicon in *N. benthamiana* plants. A mixture of *Agrobacterium* cultures containing TSWV infectious clones $L_{(+)opt}$+$M_{(–)opt}$+$SR_{(+)eGFP}$ and SlSnRK2.3/2.4-HA clones were co-infiltrated into leaf halves of *N. benthamiana* plants. A mixture of *Agrobacterium* cultures containing $L_{(+)opt}$+$M_{(–)opt}$+$SR_{(+)eGFP}$ clones and a p2300S empty vector (EV) were co-infiltrated into the other half of each leaf and used as controls. The eGFP expressed from TSWV replicon were examined and photographed under an inverted fluorescence microscope at 2.5 dpi. Bars = 800 μm. (b) Immunoblot analysis of TSWV accumulation in *N. benthamiana* leaves expressing NbSnRK2.3-/2.4-HA using eGFP and HA specific antibodies, respectively. Plant leaves without inoculation of TSWV replicon clones were used as mock controls. All experiments were repeated at least three times with similar results.

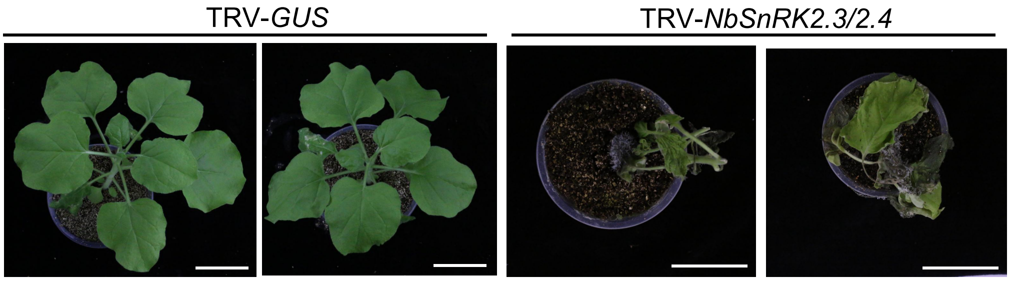

Supplementary Fig. 19 Phenotypes of *NbSnRK2.3/2.4*-silenced *N. benthamiana* plants. The mixture of *Agrobacterium* cultures containing pTRV1 and pTRV2-*GUS* or pTRV1 and pTRV2-*NbSnRK2.3/2.4* were co-infiltrated into *N. benthamiana* leaves. Photographs were taken at 21 days post TRV treatment. All experiments were repeated at least three times with similar results.

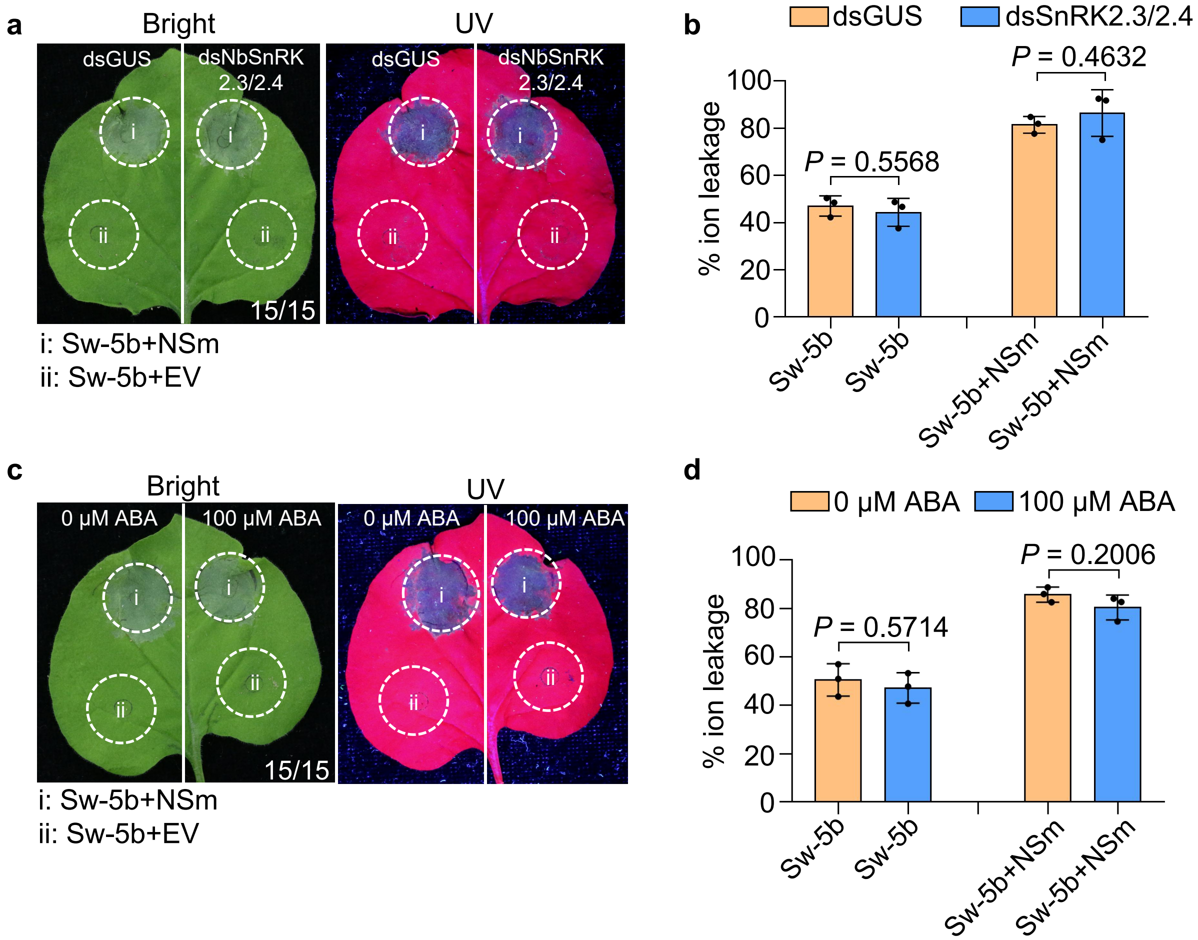

Supplementary Fig. 20 Analysis of *NbSnRK2.3/2.4* knockdown or ABA treatment on cell death induced by Sw-5b and NSm in *N. benthamiana* leaves. (a) *NbSnRK2.3/2.4* were knocked down simultaneously in *N. benthamiana* leaves by transient expression of double-stranded hairpin RNA in one half leaves, followed by (co)expression of Sw-5b or Sw-5b and NSm. Double-stranded hairpin RNA targeting GUS (dsGUS) was used as a control in another half leaves. The phenotype of hypersensitive cell death was photographed under white light and UV light at 3 dpi. (b) Knocking down *NbSnRK2.3/2.4* did not affect the ion leakage induced by Sw-5b and NSm in *N. benthamiana* leaves at 2 days post-infiltration. Data are presented as means ± s.d. (n = 3). (c) *N. benthamiana* leaves were treated with or without 100 μM ABA, followed by (co)expression of Sw-5b or Sw-5b and NSm. The phenotype of hypersensitive cell death was photographed under white light and UV light at 3 dpi. (d) ABA treatment did not affect the ion leakage induced by Sw-5b and NSm in N. benthamiana leaves at 2 days post-infiltration. Data are presented as means ± s.d. (n = 3). *P* values are indicated in the graphs. Source data are provided as a Source Data file. All experiments were repeated at least three times with similar results.

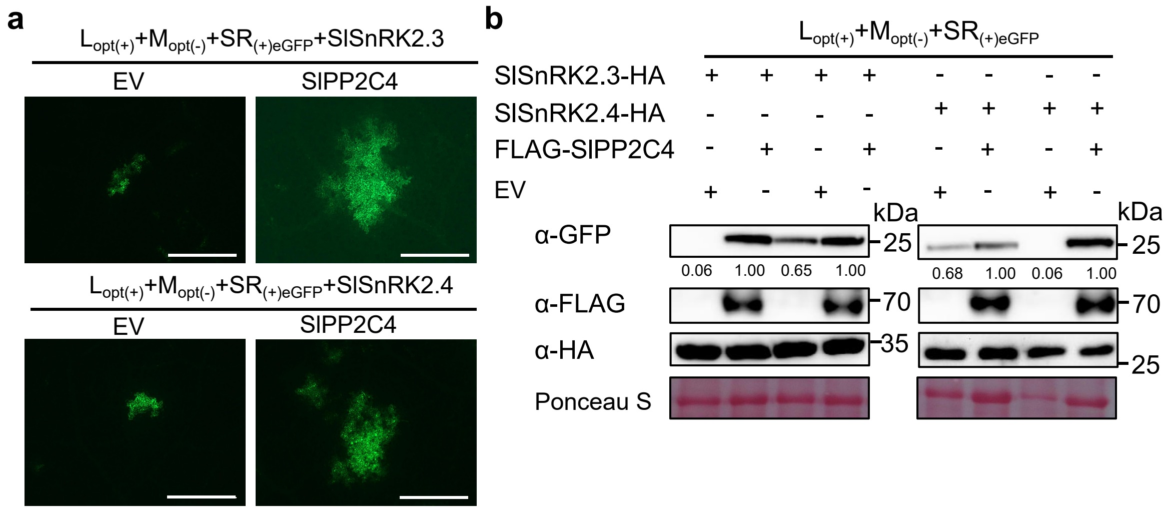

Supplementary Fig. 21 The antiviral immunity induced by SlSnRK2.3/2.4 is inhibited by SlPP2C4. (a) The TSWV infectious clones $L_{(+)opt}$+$M_{(-)opt}$+$SR_{(+)eGFP}$ were co-expressed with *SlSnRK2.3* + *SlPP2C4* (upper) or *SlSnRK2.4* + *SlPP2C4* (lower) in leaf halves of N. benthamiana. TSWV $L_{(+)opt}$+$M_{(-)opt}$+$SR_{(+)eGFP}$ were co-expressed with *SlSnRK2.3* + EV control or *SlSnRK2.4* +EV control in the other half of each leaf and used as a control. The eGFP fluorescence expressed from TSWV replicons was examined and photographed using an inverted fluorescence microscope at 2.5 dpi. Bars = 800 μm. (b) Immunoblot analysis of TSWV accumulation in panel a using GFP, FLAG and HA specific antibodies, respectively. Ponceau S staining is used to show the sample protein loadings. The protein size marker is shown in the right. All experiments were repeated at least three times with similar results.

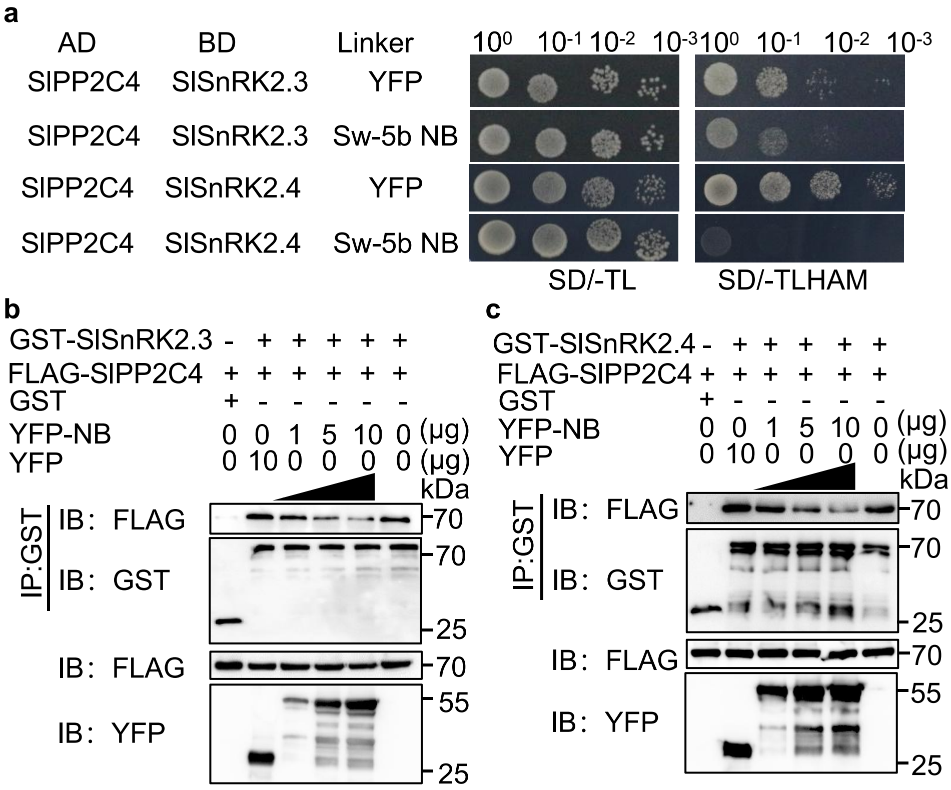

Supplementary Fig. 22 Sw-5b NB interferes with the interaction between SlPP2C4 and SlSnRK2.3/2.4. (a) Y3H assays showing the effect of Sw-5b NB on the interaction between SlPP2C4 and SlSnRK2.3 or between SlPP2C4 and SlSnRK2.4. Yeast cells were co-transformed with pGAD-SlPP2C4 and pBridge-SlSnRK2.3/2.4 +YFP or pGAD-SlPP2C4 and pBridge-SlSnRK2.3/2.4 + Sw-5b NB domain. The interactions were assayed on the SD/-TL (lacking Trp and Leu) and SD/-TLHAM (lacking Trp, Leu, His, Ade, and Met) dropout medium plates, respectively. (b and c) GST pull-down assays to assess the Sw-5b NB interference with the interaction between SlPP2C4 and SlSnRK2.3 (b) or the interaction between SlPP2C4 and SlSnRK2.4 (c) in vitro. Purified GST-SlSnRK2.3 (b) or GST-SlSnRK2.4 (c) protein was used to pull down the purified FLAG-SlPP2C4 with the increasing amount of YFP-Sw-5b NB. YFP was used as negative control. Blots were detected with FLAG, GST and YFP specific antibodies. Protein size marker is shown in the right. All experiments were repeated at least three times with similar results.

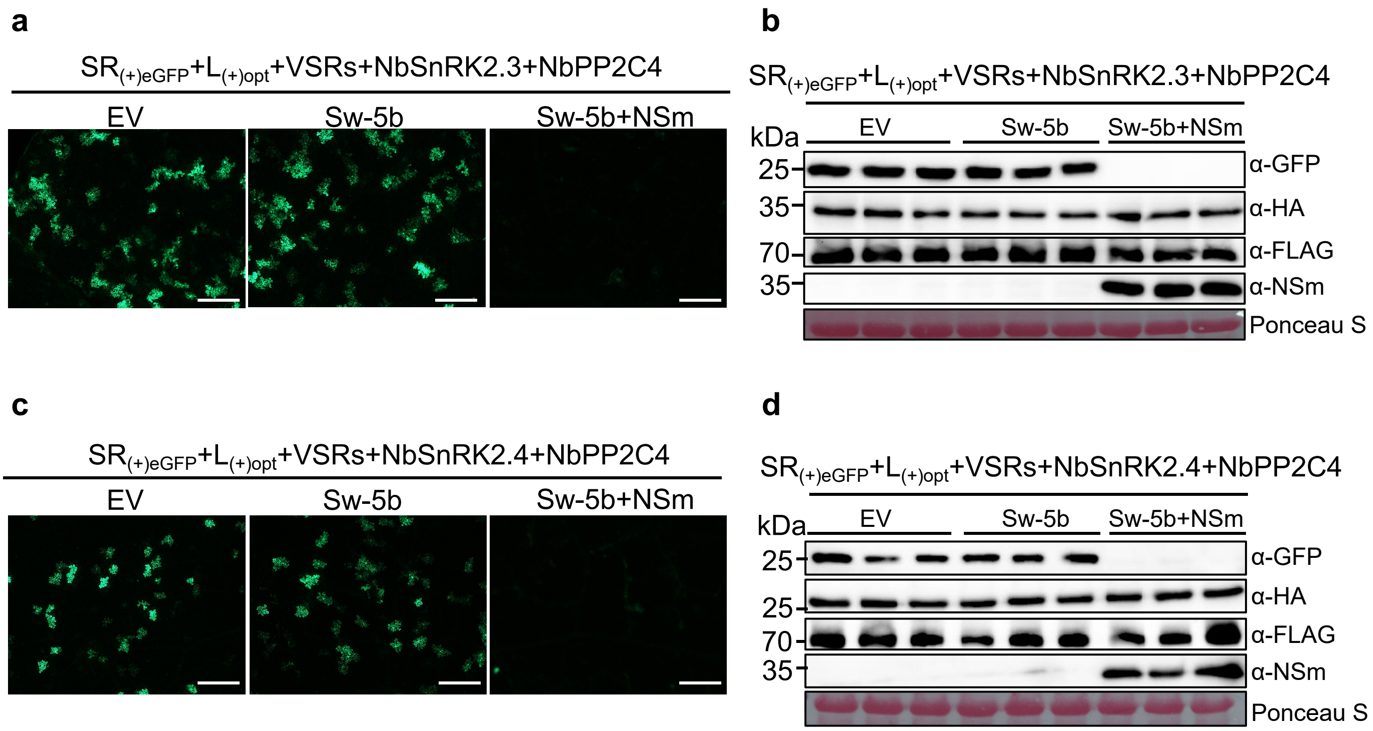

Supplementary Fig. 23 Sw-5b depresses PP2C4 repression of SnRK2.3/2.4 to activate ABA-dependent defence in the presence of NSm. (a, c) TSWV $L_{(+)opt}$ + $SR_{(+)eGFP}$ minireplicon with *NbSnRK2.3* and *NbPP2C4* (a) or *NbSnRK2.4* and *NbPP2C4* (c) were co-expressed with pCambia2300 empty vector (EV) control, Sw-5b or Sw-5b+NSm in *N. benthamiana* leaves. These combinations were co-expressed with three viral RNA silencing suppressors (VSRs). The eGFP fluorescence expressed from the TSWV $SR_{(+)eGFP}$ minireplicon was examined and photographed under an inverted fluorescence microscope at 2.5 dpi. Bars = 600 μm. (b, d) Immunoblot analyses of TSWV $SR_{(+)eGFP}$ minireplicon accumulation shown in panel a and c, respectively, using GFP-, HA-, FLAG- and NSm-specific antibodies. Ponceau S staining is used to show the sample protein loadings. All experiments were repeated at least three times with similar results.

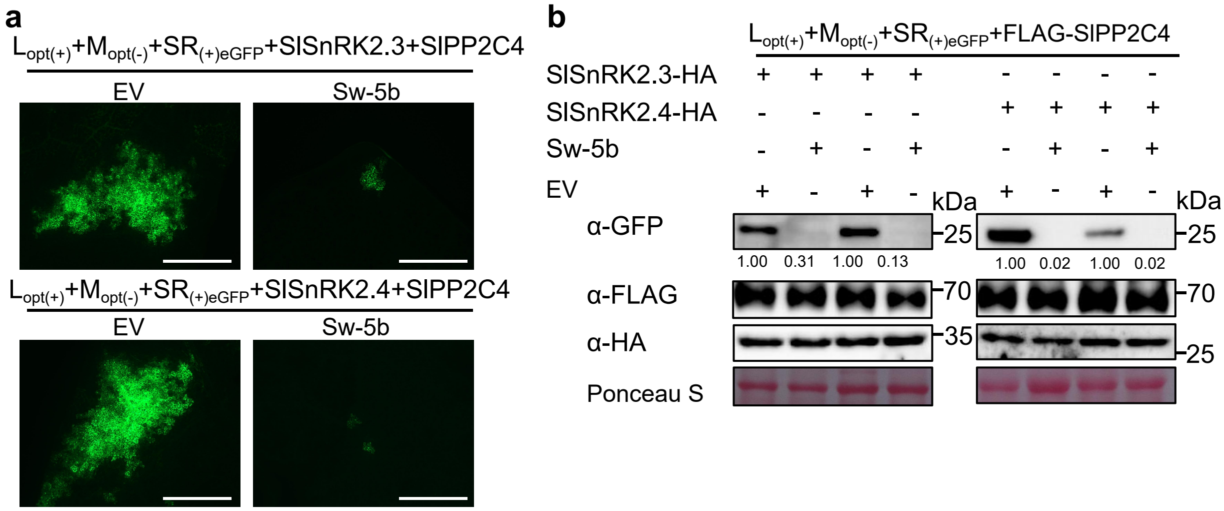

Supplementary Fig. 24 Sw-5b depresses the inhibition of *SlPP2C4* on *SlSnRK2.3/2.4* to active antiviral immunity. (a) The TSWV infectious clones $L_{(+)opt}$+$M_{(-)opt}$+$SR_{(+)eGFP}$ were co-expressed with Sw-5b, *SlSnRK2.3* and *SlPP2C4* (upper) or Sw-5b, *SlSnRK2.4* and *SlPP2C4* (lower) in leaf halves of N. benthamiana plant. Those combinations were also co-expressed with a p2300S empty vector (EV) control in the other half of each leaf and used as a control. The eGFP fluorescence expressed from TSWV replicon was examined and photographed using an inverted fluorescence microscope at 2.5 dpi. Bars = 200 μm. (b) Immunoblot analysis of TSWV accumulation in panel a using GFP, FLAG and HA specific antibodies, respectively. Ponceau S staining is used to show the sample protein loadings. Protein size marker is shown in the right. All experiments were repeated at least three times with similar results.

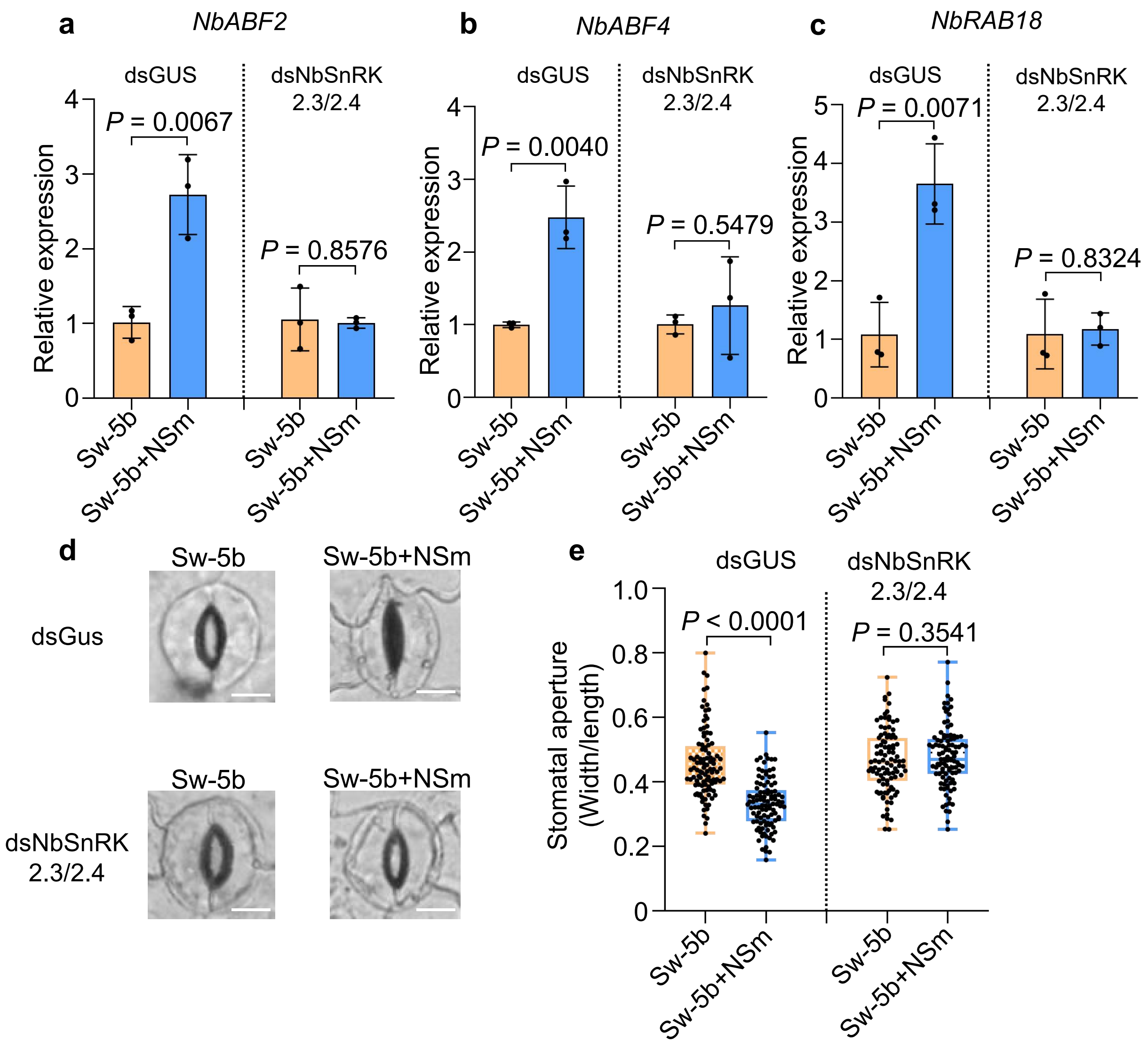

Supplementary Fig. 25 *SnRK2.3/2.4* is required for Sw-5b and NSm to activate ABA response genes and induce stomatal closure. (a-c) Relative expression levels of ABA responsive genes *NbABF2*, *NbABF4*, and *NbRAB18* in *NbSnRK2.3/2.4*-silenced or dsGUS-treated control *N. benthamiana* leaves by (co)expressing Sw-5b or Sw-5b+NSm. *N. benthamiana* plants were treated with hairpin RNA construct targeting *NbSnKR2.3/2.4* (ds*NbSnKR2.3/2.4*) or with dsGUS control. *NbActin* was used as the internal reference gene. Data are presented as means ± s.d. (n = 3). (d) Co-expression of Sw-5b and NSm failed to induce stomatal closure in *NbSnRK2.3/2.4*-silenced *N. benthamiana* leaves. *N. benthamiana* leaves treated with hairpin RNA construct targeting *NbSnKR2.3/2.4* (ds*NbSnKR2.3/2.4*) or with dsGUS control were (co)expressed with Sw-5b, or Sw-5b, and NSm via agroinfiltration. Stomatal aperture

was measured at 24 hpai. Bars = 10 μm. (e) Statistical analysis results of stomatal aperture in *NbSnRK2.3/2.4*-silenced or dsGUS control leaves (co)expressed with Sw-5b, or Sw-5b and NSm. Data are shown as the box plots with the interquartile range as the upper and lower confines, minima and maxima as whiskers, and the median as a solid line (n = 100, the number of stomata). The exact *P* values are indicated in the graphs or Source data (two-sided Student's t-test). Source data are provided as a Source Data file.

Supplementary Table 1: The interacting proteins of Sw-5b NB selected by yeast two-hybrid screening of cDNA library from *Nicotiana benthamiana*.

Number	NCBI Reference Sequence	Functional annotation	Frequency of occurrence	Gene ID
**1**	XP_016437307	PP2C4	3	NbS00038103g0004
**2**	XM_016607559	SGT1	2	NbS00031070g0015
**3**	interacting protein 3		6	
**……**	**……**	**……**	**……**	**……**
**35**	interacting protein 35		1	

Supplementary Table 2: Primers used in this study.

Primers	Sequences	Usage
FLAG-SlPP2C4-F	CGCGAGCTCGGTACCATGGATTACAAGGATGACGACG ATAAGATGGAAGAGATGTTTCCA	Cloning
FLAG-SlPP2C4-R	GGCCGGCAGGTCGACTCTAGATTATGTTTTCTTCTTGA ATTTC	Cloning
FLAG-NbPP2C4-F	TCGCGAGCTCGGTACCATGGATTACAAGGATGACGAC GATAAGATGGAAGAGATGTTAC	Cloning
FLAG-NbPP2C4-R	GGCAGGTCGACTCTAGACTACGTTTTCTTCTTGAA	Cloning
NbSnRK2.3-HA-F	CTTTCGCGAGCTCGGTACCATGGAATACGCATCTGG	Cloning
NbSnRK2.3-HA-R	CTTGCATGCCTGCAGTCAAGCGTAATCTGGAACATCG TATGGGTACATGGCATAGACAA	Cloning
NbSnRK2.4-HA-F	CTTTCGCGAGCTCGGTACCATGGATCGAGGTCTG	Cloning
NbSnRK2.4-HA-R	CTTGCATGCCTGCAGTCAAGCGTAATCTGGAACATCG TATGGGTACATTGCATATATGA	Cloning
SlSnRK2.3-HA-F	TTTCGCGAGCTCGGTACCATGGATCGGACGGCAGT	Cloning
SlSnRK2.3-HA-R	CTTGCATGCCTGCAGTCAAGCGTAATCTGGAACATCG TATGGGTACATTGCATAGACAA	Cloning
SlSnRK2.4-HA-F	TTCGCGAGCTCGGTACCATGGAGCAACTGCT	Cloning
SlSnRK2.4-HA-R	CTTGCATGCCTGCAGTCAAGCGTAATCTGGAACATCG TATGGGTACATTGCATATATGAT	Cloning
GST-NbSnRK2.3-F	CGTGCATCTGTTGGATCCATGGAATACGCATCTGGAG GAGA	Cloning
GST-NbSnRK2.3-R	TCAGTCACGATGAATTCTTACATGGCATAGACAATC	Cloning
GST-NbSnRK2.4-F	GTGCATCTGTTGGATCCATGGATCGAGGTCTGATTCC	Cloning
GST-NbSnRK2.4-R	GTCAGTCACGATGAATTCTCACATTGCATATATGATCT	Cloning
GST-SlSnRK2.3-F	TCGTGCATCTGTTGGATCCATGGATCGGACGGCAGTG A	Cloning
GST-SlSnRK2.3-R	TCAGTCACGATGAATTCTTACATTGCATAGACAATC	Cloning
GST-SlSnRK2.4-F	GTGCATCTGTTGGATCCATGGAGCAACTGCTAGTCT	Cloning
GST-SlSnRK2.4-R	TCAGTCACGATGAATTCTCACATTGCATATATG	Cloning
His-FLAG-SlPP2C4-F	CCGCGCGGCAGCCATATGGATTACAAGGATGACGAC GATAAGATGGAAGAGATGTTT	Cloning
His-FLAG-SlPP2C4-R	CGACGGAGCTCGAATTCTCATGTTTTCTTCTTGAA	Cloning
His-FLAG-NbPP2C4-F	GCGCGGCAGCCATATGGATTACAAGGATGACGACGAT AAGATGGAAGAGATGTTACC	Cloning
His-FLAG-NbPP2C4-R	CGGAGCTCGAATTCTCACTACGTTTTCTTCTT	Cloning
His-YFP-F	CAAATGGGTCGCGGATCCATGGTGAGCAAGGGCGAG	Cloning
His-YFP-R	TCGACGGAGCTCGAATTCAGATCTGTACAGCTCGT	Cloning
His-YFP-NB-F	CGCGCGGCAGCCATATGATGGTGAGCAAGGGCGAGG	Cloning
His-YFP-NB-R	GGAGCTCGAATTCGGATCCTTATGTTGTGAGGAATG	Cloning

Supplementary Table 2 continued

Primers	Sequences	Usage
AD-SlPP2C4-F	GATTACGCTCATATGATGGAAGAGATGTTTCCA	Cloning
AD-SlPP2C4-R	GAGCTCGATGGATCCTTATGTTTTCTTCTTGAA	Cloning
AD-NbPP2C4-F	TACGCTCATATGATGTCTGGTAAATGGACCCACTTG	Cloning
AD-NbPP2C4-R	GAGCTCGATGGATCCCTACGTTTTCTTCTTGAA	Cloning
BK-SlSnRK2.3-F	AGGAGGACCTGCATATGATGGATCGGACGGCAGT	Cloning
BK-SlSnRK2.3-R	CGCTGCAGGTCGACGGATCCTTACATTGCATAGACAA	Cloning
BK-SlSnRK2.4-F	AGGAGGACCTGCATATGATGGAGCAACTGCTAGTC	Cloning
BK-SlSnRK2.4-R	CGCTGCAGGTCGACGGATCCTCACATTGCATATATGAT	Cloning
BK-NbSnRK2.3-F	GAGGAGGACCTGCATATGATGGAATACGCATCTGGAGG	Cloning
BK-NbSnRK2.3-R	CGGATCCCCGGGAATTCTTACATGGCATAGACAATCT	Cloning
BK-NbSnRK2.4-F	AGGAGGACCTGCATATGATGGATCGAGGTCTGATTCC	Cloning
BK-NbSnRK2.4-R	CGACGGATCCCCGGGAATTCTCACATTGCATATATGAT	Cloning
TRV-NbSnRK2.3/2.4-F1	TTACCGAATTCTCTAGAAGAATGTCGTCATCTAAT	Cloning
TRV-NbSnRK2.3/2.4-R1	CCGGAAATCTTTTGGTTCATCAGGGTCACTCTCCAA	Cloning
TRV-NbSnRK2.3/2.4-F2	TTGGAGAGTGACCCTGATGAACCAAAAGATTTCCGG	Cloning
TRV-NbSnRK2.3/2.4-R2	ACGCGTGAGCTCGGTACCCCAACTGGTGGTACTG	Cloning
BD-Sw-5b-NB-F	GAGAAAGGTGGCGGCCGCACCATTAAAACATCTGC	Cloning
BD-Sw-5b-NB-R	ATCAGCCCGAAGATCTCTATGTTGTGAGGAAT	Cloning
BD-NbSnRK2.3-NB-F1	TTCCCGGGGATCCGTATGGAATACGCATCTGGAGG	Cloning
BD-NbSnRK2.3-NB-R1	GAATTAGCTTGGCTGCAGTTACATGGCATAGACAAT	Cloning
BD-NbSnRK2.4-NB-F1	TTCCCGGGGATCCGTATGGATCGAGGTCTGATTCC	Cloning
BD-NbSnRK2.4-NB-R1	GAATTAGCTTGGCTGCAGTCACATTGCATATATGATC	Cloning
BD-SlSnRK2.3-NB-F1	TTCCCGGGGATCCGTATGGATCGGACGGCAGTG	Cloning
BD-SlSnRK2.3-NB-R1	GGAATTAGCTTGGCTGCAGTTACATTGCATAGACAAT	Cloning
BD-SlSnRK2.4-NB-F	TTCCCGGGGATCCGTATGGAGCAACTGCTAGTC	Cloning
BD-SlSnRK2.4-NB-R	GGAATTAGCTTGGCTGCAGTCACATTGCATATATGA	Cloning

Supplementary Table 2 continued

Primers	Sequences	Usage
BD-YFP-F	AAAGGTGGCGGCCGCAATGGTGAGCAAGGGC	Cloning
BD-YFP-R	GAGATCAGCCCGAAGATCTTTAAGATCTGTACA	Cloning
TRV2-SlPP2C4-F	TACCGAATTCTCTAGAGGGAAAATTGATATATGAT	Cloning
TRV2-SlPP2C4-R	CGCGTGAGCTCGGTACCTCAGTTGGTGAAATCAAA	Cloning
TRV2-SlABA2-F	TTACCGAATTCTCTAGAGACTGATACTTCTCTTC	Cloning
TRV2-SlABA2-R	ACGCGTGAGCTCGGTACCAGACAACCCGGCATTA	Cloning
TRV2-NbABA2-F	TTACCGAATTCTCTAGACGGTGATGACCAAAGTGC	Cloning
TRV2-NbABA2-R	ACGCGTGAGCTCGGTACCCCAGCAATAGCACTTG	Cloning
dsNbSnRK2.3/2.4-F1	GCGAGCTCGGTACCCCTGCAGATCTCATGGATG	Cloning
dsNbSnRK2.3/2.4-R1	TCTTACACATTTGGATCCCGTCCATTAAATCTGC	Cloning
dsNbSnRK2.3/2.4-F2	TTGCAGGTATTTCTAGACGTCCATTAAATCTGCC	Cloning
dsNbSnRK2.3/2.4-R2	TTGCATGCCTGCAGCCGGCAGATCTCATGGAT	Cloning
cLUC-NB-F	GGGGTACCATGCCATTAAAACATCTGC	Cloning
cLUC-NB-R	CGGGATCCTCATGTTGTGAGGAATGG	Cloning
nLUC-NbPP2C4-F	GGGACGAGCTCGGTACCATGGAAGAGATGTTAC	Cloning
nLUC-NbPP2C4-R	TACGAGATCTGGTCGACCGTTTTCTTCTTGAATT	Cloning
nLUC-SlPP2C4-F	ATTCGAGCTCGGTACCATGGAAGAGATGTTTCC	Cloning
nLUC-SlPP2C4-R	ACGCGTCGACTGTTTTCTTCTTGAA	Cloning
sgRNA-NbPP2C4-F	ATTGGTTCTCTAGTGGATGAGCG	Mutagenesis
sgRNA-NbPP2C4-R	AAACCGCTCATCCACTAGAGAAC	Mutagenesis
sgRNA-NbPP2C4-F	ATGGAAGAGATGTTACCAACAC	Genotyping
sgRNA-NbPP2C4-R	CCTGAGAGCCTCCATGTCCATC	Genotyping
qPCR-NbABF2-F	CCGCTCTGTTGGGCACATTA	qPCR
qPCR-NbABF2-R	AAGCCTGCTTCCTTGCTCGT	qPCR
qPCR-NbABF4-F	CACCTTGGGAGAAATGACG	qPCR
qPCR-NbABF4-R	GGGTTTTGAGTTGCTTGTT	qPCR
qPCR-NbRAB18-F	GGTGGTTGCTGCTCATGAAAC	qPCR
qPCR-NbRAB18-R	AGCTCCATCTCGTGCACAGTT	qPCR
qPCR-SlABF2-F	GGCAAGGCTCTTTGACTCTAC	qPCR
qPCR-SlABF2-R	ACTATTTCCATCCTTCCCACC	qPCR

Supplementary Table 2 continued

Primers	Sequences	Usage
qPCR-SlRAB18-F	ACGCCAGCATGGTACTCTTG	qPCR
qPCR-SlRAB18-R	TCACCTTGTCCTTGATCCCT	qPCR
qPCR-SlTAS14-F	ACGGCAATCAAGACCAAAT	qPCR
qPCR-SlTAS14-R	CCTTCTTTCTCCTCCCACC	qPCR
qPCR-SlABA2-F	CTGCCAGGAATGCTAACT	qPCR
qPCR-SlABA2-R	TATCTGAAGACCCGAAGG	qPCR
qPCR-NbABA2-F	CCTGGGAATGAAACACGC	qPCR
qPCR-NbABA2-R	CCCAATTCAGCAGCCACA	qPCR
