# Supplementary material for: A plant NLR receptor employs ABA central regulator PP2C-SnRK2 to activate antiviral immunity
Source: Nat Commun. 2024 Apr 13;15:3205. doi: 10.1038/s41467-024-47364-8 (PMC11016096; doi:10.1038/s41467-024-47364-8)
Supplement: Supplementary file 3 — Reporting Summary [file 41467_2024_47364_MOESM3_ESM.pdf]

nature portfolio

Corresponding author(s): Xiaorong Tao

Last updated by author(s): Dec 12, 2023

Reporting Summary

Nature Portfolio wishes to improve the reproducibility of the work that we publish. This form provides structure for consistency and transparency in reporting. For further information on Nature Portfolio policies, see our Editorial Policies and the Editorial Policy Checklist.

Statistics

For all statistical analyses, confirm that the following items are present in the figure legend, table legend, main text, or Methods section.

n/a	Confirmed	
☐	☒	The exact sample size (n) for each experimental group/condition, given as a discrete number and unit of measurement
☐	☒	A statement on whether measurements were taken from distinct samples or whether the same sample was measured repeatedly
☐	☒	The statistical test(s) used AND whether they are one- or two-sided<br>*Only common tests should be described solely by name; describe more complex techniques in the Methods section.*
☒	☐	A description of all covariates tested
☐	☒	A description of any assumptions or corrections, such as tests of normality and adjustment for multiple comparisons
☐	☒	A full description of the statistical parameters including central tendency (e.g. means) or other basic estimates (e.g. regression coefficient) AND variation (e.g. standard deviation) or associated estimates of uncertainty (e.g. confidence intervals)
☐	☒	For null hypothesis testing, the test statistic (e.g. F, t, r) with confidence intervals, effect sizes, degrees of freedom and P value noted<br>*Give P values as exact values whenever suitable.*
☒	☐	For Bayesian analysis, information on the choice of priors and Markov chain Monte Carlo settings
☒	☐	For hierarchical and complex designs, identification of the appropriate level for tests and full reporting of outcomes
☒	☐	Estimates of effect sizes (e.g. Cohen's d, Pearson's r), indicating how they were calculated

Our web collection on statistics for biologists contains articles on many of the points above.

Software and code

Policy information about availability of computer code

Data collection	Gene expression (qRT-PCR): ABI Prism 7500 Fast Real-Time PCR system<br>Western blots: Bio-Rad ChemiDocTM Touch Imaging System<br>Confocal microscopy : Carl-zeiss LSM 710 and Zen blue software<br>Split-luciferase complementation: VILBER Fusion FX7 imaging apparatus and FusionCapt Advance (FX7)<br>Phytohormone quantification: Triple Quadrupole Xevo TQ-S System<br>Photographing of plant: Canon EOS 70D digital camera
Data analysis	Gene expression (qRT-PCR): Graphpad Prism software (9.0.0)<br>Protein quantification: Image Lab (5.2.1) and ImageJ (1.50i) software<br>Confocal microscopy: Carl-zeiss Zen blue software<br>Split-luciferase complementation: VILBER Fusion FX7 imaging apparatus and FusionCapt Advance (FX7)<br>Statistics and graph production: Graphpad Prism software (9.0.0)<br>Phytohormone quantification: Triple Quadrupole Xevo TQ-S System

For manuscripts utilizing custom algorithms or software that are central to the research but not yet described in published literature, software must be made available to editors and reviewers. We strongly encourage code deposition in a community repository (e.g. GitHub). See the Nature Portfolio guidelines for submitting code & software for further information.

Data

Policy information about availability of data

All manuscripts must include a data availability statement. This statement should provide the following information, where applicable:

- Accession codes, unique identifiers, or web links for publicly available datasets
- A description of any restrictions on data availability
- For clinical datasets or third party data, please ensure that the statement adheres to our policy

All data are available within this article and its supplementary files. All constructs and transgenic plants are available upon request. The PP2C4 and SnRK2 gene sequences are available from the Sol Genomics Network (https://www.sgn.cornell.edu/) using the accession numbers provided in this article. Source data are provided with this paper.

Research involving human participants, their data, or biological material

Policy information about studies with human participants or human data. See also policy information about sex, gender (identity/presentation), and sexual orientation and race, ethnicity and racism.

Reporting on sex and gender	N/A
Reporting on race, ethnicity, or other socially relevant groupings	N/A
Population characteristics	N/A
Recruitment	N/A
Ethics oversight	N/A

Note that full information on the approval of the study protocol must also be provided in the manuscript.

Field-specific reporting

Please select the one below that is the best fit for your research. If you are not sure, read the appropriate sections before making your selection.

☒ Life sciences ☐ Behavioural & social sciences ☐ Ecological, evolutionary & environmental sciences

For a reference copy of the document with all sections, see nature.com/documents/nr-reporting-summary-flat.pdf

Life sciences study design

All studies must disclose on these points even when the disclosure is negative.

Sample size	The sample size and statistical analyses are described in the relevant Figure legends. No statistical methods were used to predetermine sample size. Sample size was determined based on previous publications.<br>Gene expression analysis (https://www.nature.com/articles/s41586-021-03987-1#ref-CR40)<br>Seed germination and root elongation assays (https://onlinelibrary.wiley.com/doi/10.1111/jipb.13257)<br>Split-luciferase complementation assay (https://academic.oup.com/plphys/article/146/2/323/6107098?login=true)<br>Phytohormone measurement (https://academic.oup.com/plphys/article/177/2/476/6117035?login=true)
Data exclusions	No data were excluded from the analyses.
Replication	Each experiment was repeated at least three times. Results were reproducible with the same trend.
Randomization	Allocation of test plants used in our study was random. There was no targeted selection of individual plants for specific treatments.
Blinding	Blinding was not used in our study. In plant, biology blinded/double-blinded studies are uncommon.

Reporting for specific materials, systems and methods

We require information from authors about some types of materials, experimental systems and methods used in many studies. Here, indicate whether each material, system or method listed is relevant to your study. If you are not sure if a list item applies to your research, read the appropriate section before selecting a response.

Materials & experimental systems

n/a	Involved in the study
☐	☒ Antibodies
☒	☐ Eukaryotic cell lines
☒	☐ Palaeontology and archaeology
☒	☐ Animals and other organisms
☒	☐ Clinical data
☒	☐ Dual use research of concern
☐	☒ Plants

Methods

n/a	Involved in the study
☒	☐ ChIP-seq
☒	☐ Flow cytometry
☒	☐ MRI-based neuroimaging

Antibodies

Antibodies used

anti-FLAG-HRP (Sigma-Aldrich, Cat. # A8592; clone M2; 1:10000)
anti-HA (Abcam, Cat. # ab18181; clone C5, 1:2000)
anti-GST (Sigma-Aldrich Cat. # SAB1305539; clone 9AT106; 1:5000)
anti-GFP (Sigma-Aldrich Cat. # SAB4301138; 1:10000)
Goat Anti-Rabbit IgG HRP (Sigma-Aldrich Cat # A0545; 1:10000)
Goat Anti-mouse IgG HRP (Sigma-Aldrich Cat # A4416; 1:10000)
N and NSm proteins were expressed and purified from E.coli respectively, antibodies against N (1:5000) and NSm (1:5000) proteins were generated in rabbits.

Validation

Validation statements and experiments can be obtained from the following websites:
anti-FLAG-HRP (https://www.sigmaaldrich.cn/CN/zh/product/sigma/a8592)
anti-HA (https://www.abcam.cn/ha-tag-antibody-hac5-ab18181.html)
anti-GST (https://www.sigmaaldrich.cn/CN/zh/product/sigma/sab1305539)
anti-YFP (https://www.sigmaaldrich.cn/CN/zh/product/sigma/sab4301138)
Goat Anti-Rabbit IgG HRP (https://www.sigmaaldrich.cn/CN/zh/product/sigma/a0545)
Goat Anti-Mouse IgG HRP (https://www.sigmaaldrich.cn/CN/zh/product/sigma/a4416)

Dual use research of concern

Policy information about dual use research of concern

Hazards

Could the accidental, deliberate or reckless misuse of agents or technologies generated in the work, or the application of information presented in the manuscript, pose a threat to:

No	Yes
☒	☐ Public health
☒	☐ National security
☒	☐ Crops and/or livestock
☒	☐ Ecosystems
☒	☐ Any other significant area

Experiments of concern

Does the work involve any of these experiments of concern:

No	Yes
☒	☐ Demonstrate how to render a vaccine ineffective
☒	☐ Confer resistance to therapeutically useful antibiotics or antiviral agents
☒	☐ Enhance the virulence of a pathogen or render a nonpathogen virulent
☒	☐ Increase transmissibility of a pathogen
☒	☐ Alter the host range of a pathogen
☒	☐ Enable evasion of diagnostic/detection modalities
☒	☐ Enable the weaponization of a biological agent or toxin
☒	☐ Any other potentially harmful combination of experiments and agents
