# Supplementary material for: A plant NLR receptor employs ABA central regulator PP2C-SnRK2 to activate antiviral immunity
Source: Nat Commun. 2024 Apr 13;15:3205. doi: 10.1038/s41467-024-47364-8 (PMC11016096; doi:10.1038/s41467-024-47364-8)

Fig. 1c

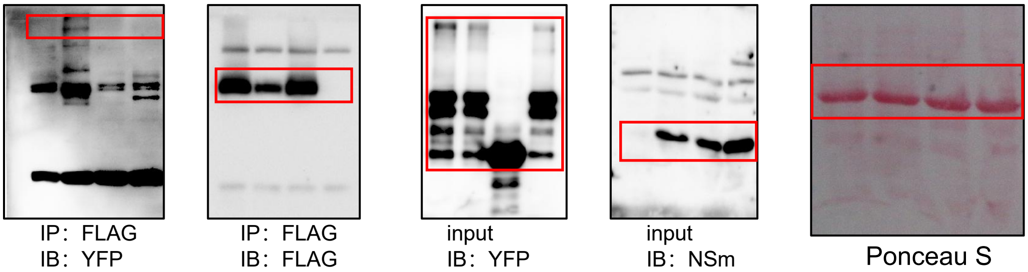

Fig. 1j

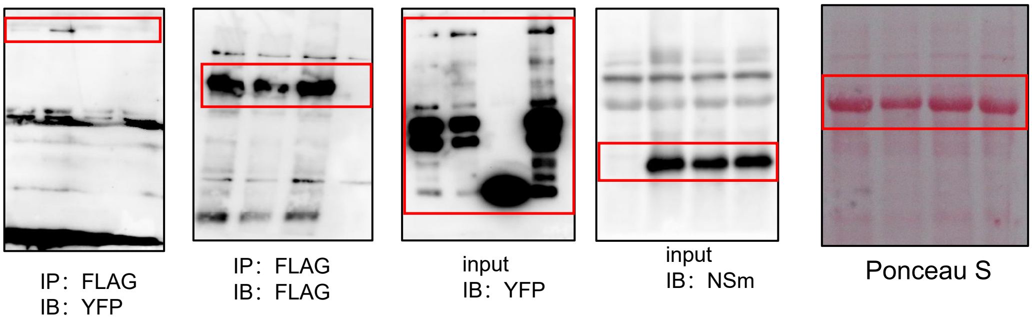

Fig. 2b

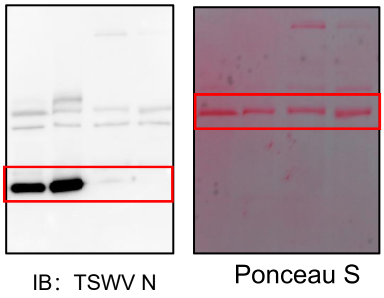

Fig. 2d

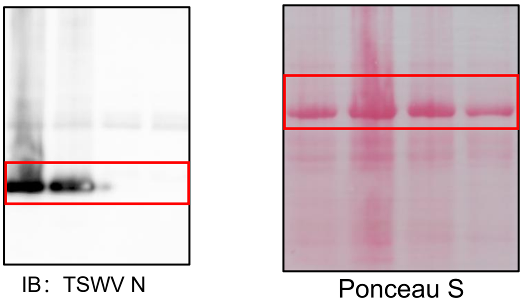

Fig. 2h

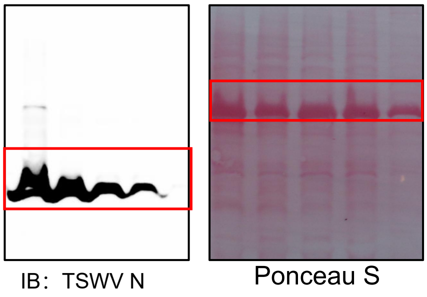

Fig. 2k

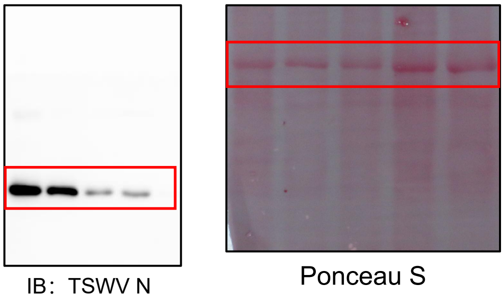

Fig. 3c

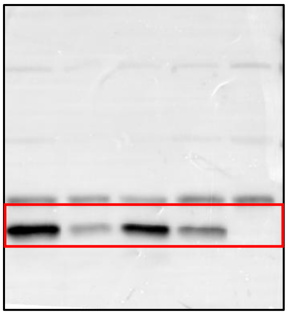

IB：GFP

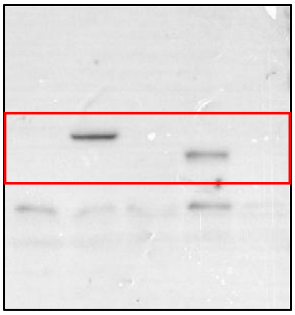

IB：HA

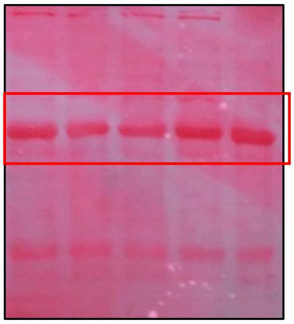

Ponceau S

Fig. 3f

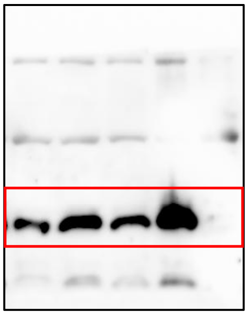

IB：GFP

Fig. 3f

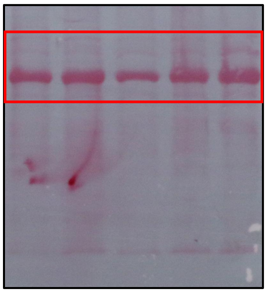

Ponceau S

Fig. 3i

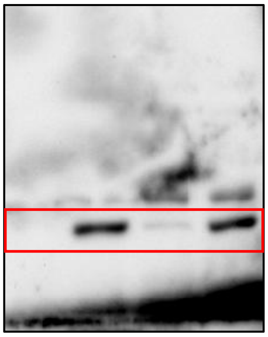

IB：TSWV N

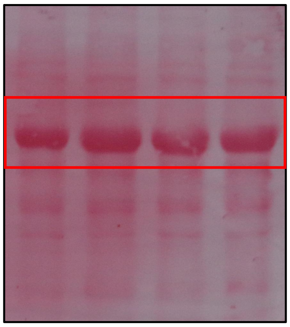

Ponceau S

Fig. 3k

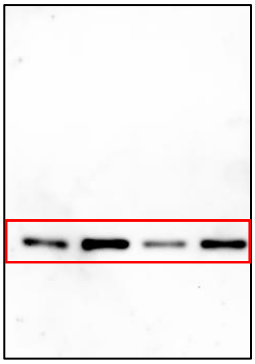

IB：GFP

Fig. 3k

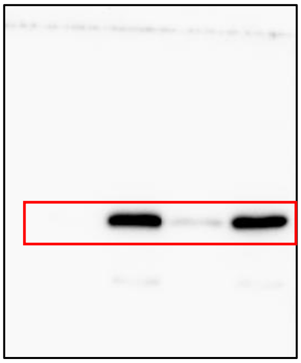

IB：GFP

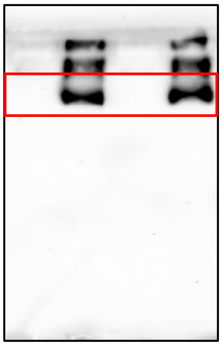

IB：FLAG

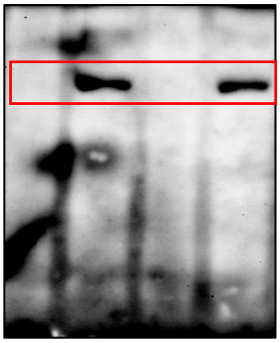

IB：FLAG

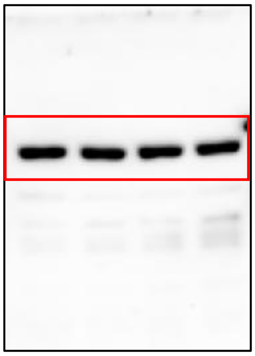

IB：HA

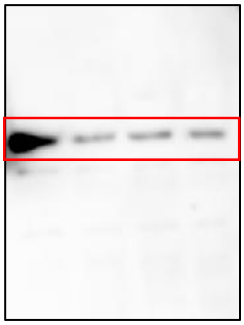

IB：HA

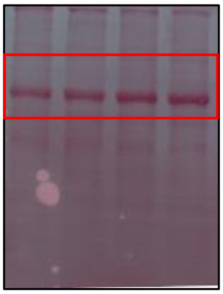

Ponceau S

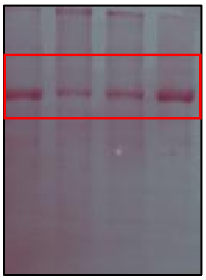

Ponceau S

Fig. 4b

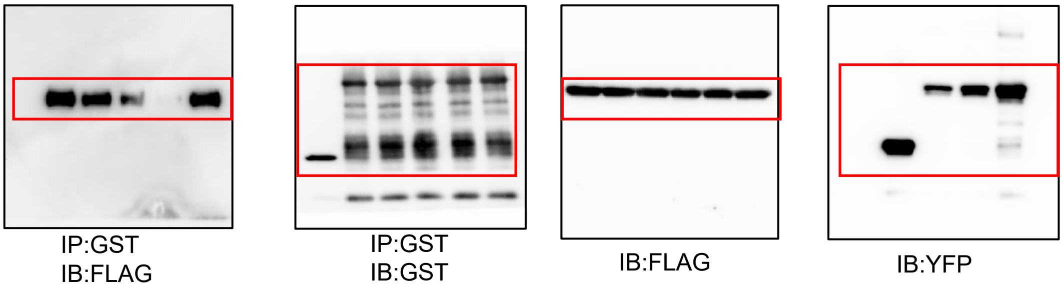

Fig. 4c

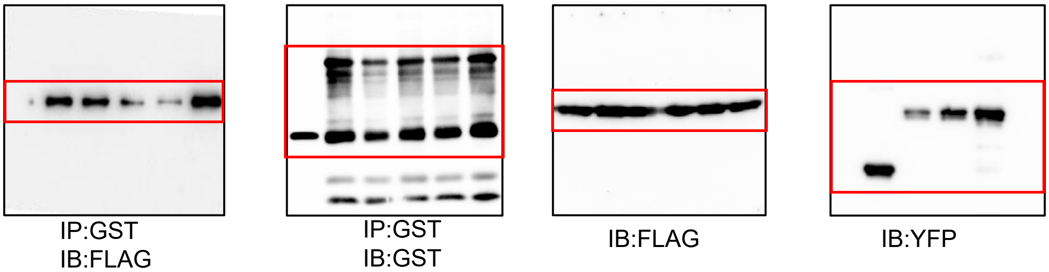

Fig. 4e

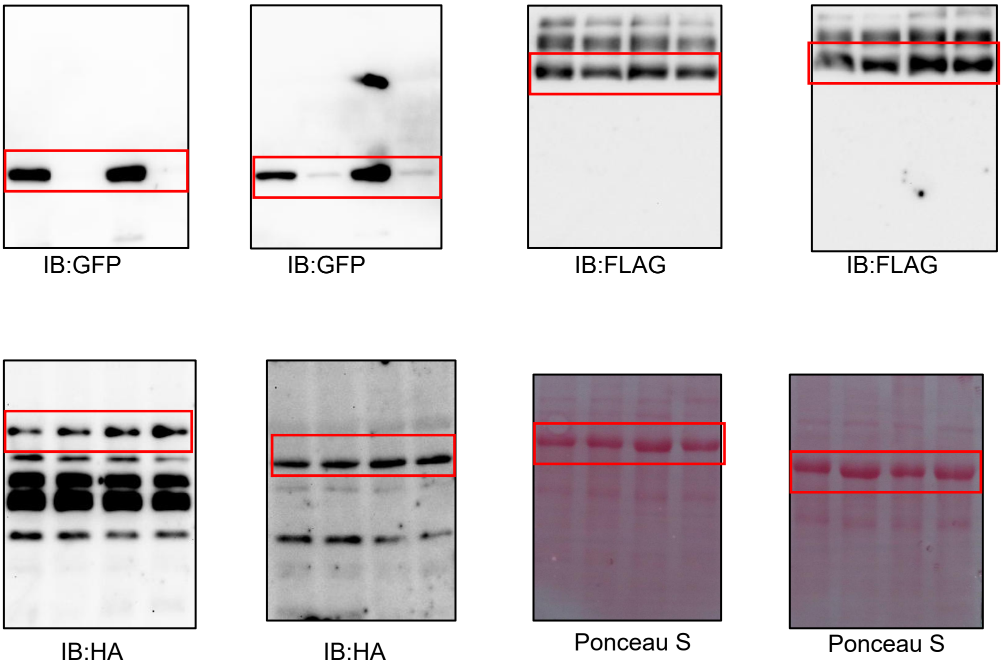

Supplementary Fig. 4a

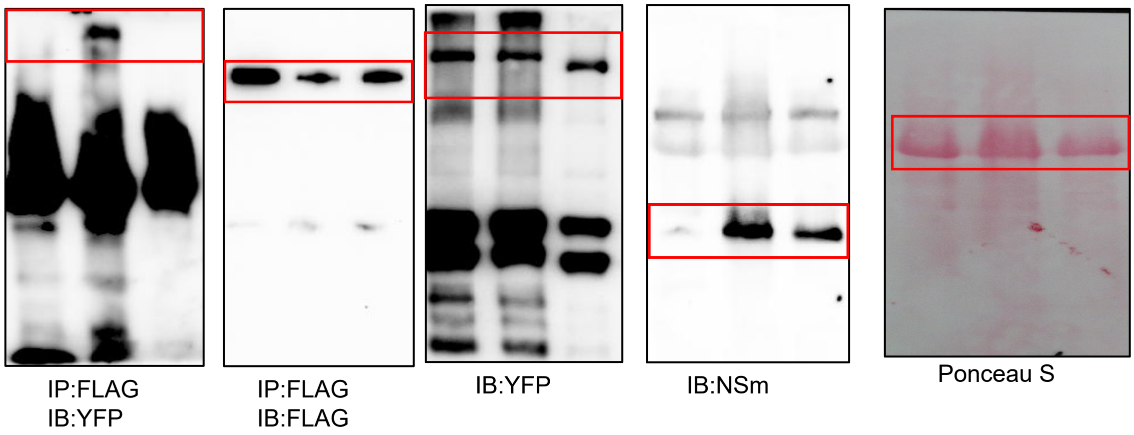

Supplementary Fig. 4b

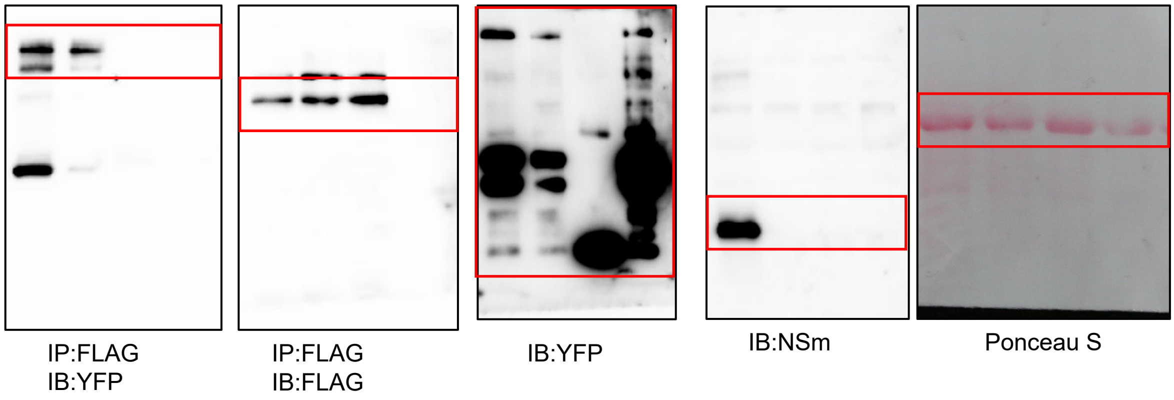

Supplementary Fig. 7

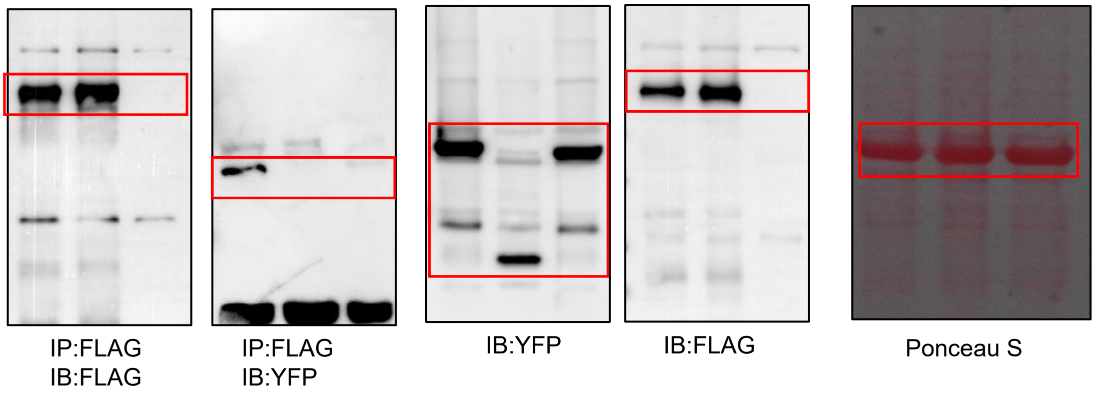

Supplementary Fig. 10c

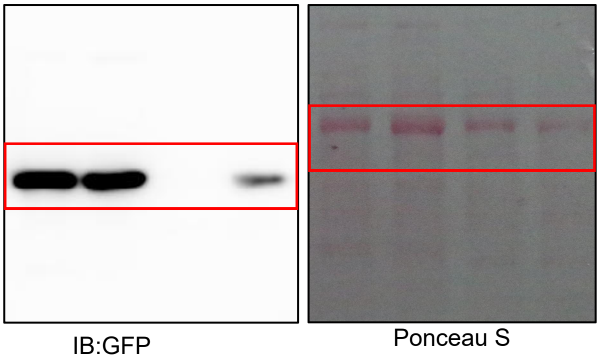

Supplementary Fig. 14b

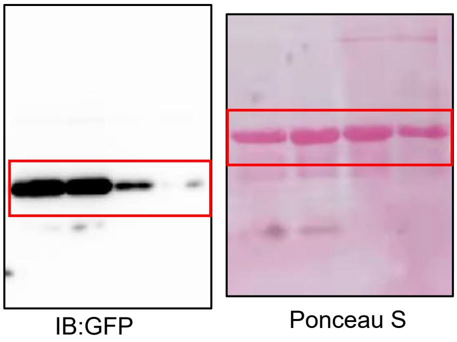

Supplementary Fig. 15c

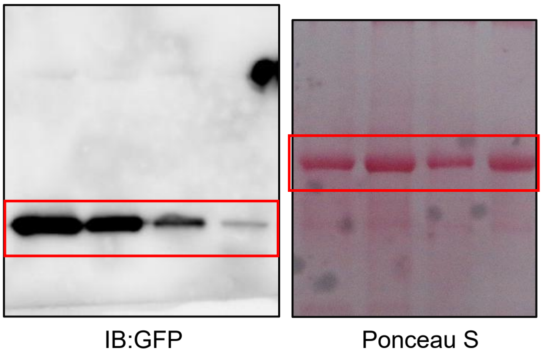

Supplementary Fig. 15d

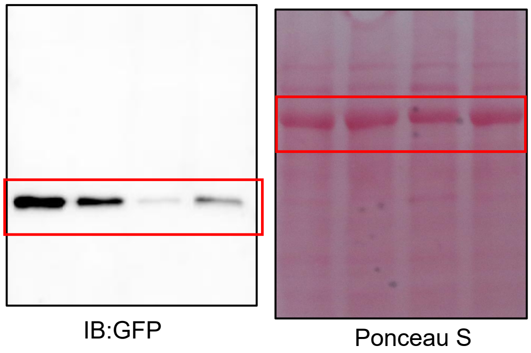

Supplementary Fig. 18b

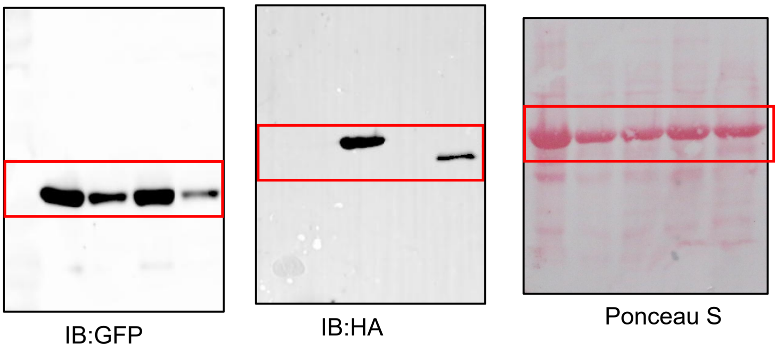

Supplementary Fig. 21b

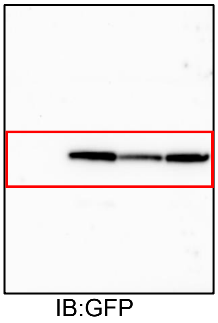

Supplementary Fig. 21b

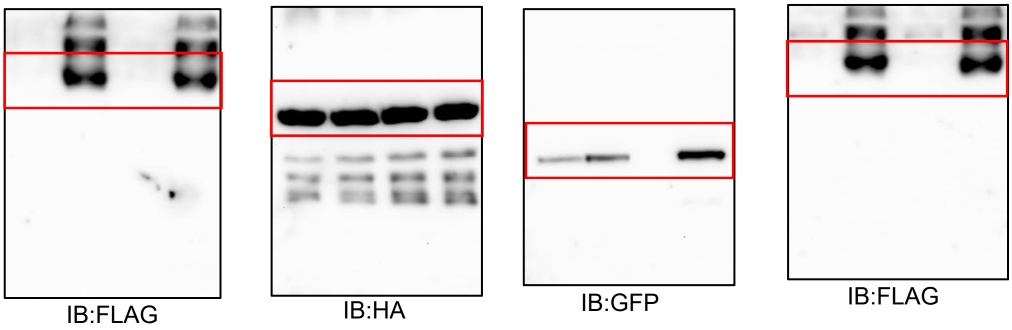

Supplementary Fig. 21b

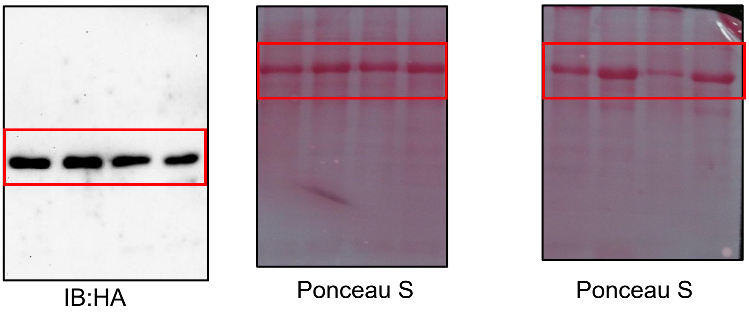

Supplementary Fig. 22b

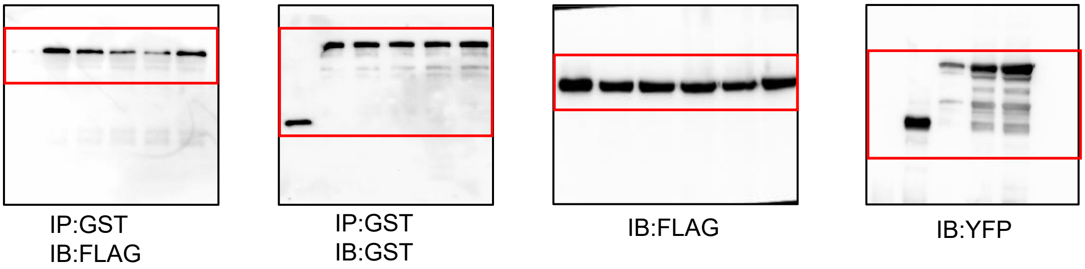

Supplementary Fig. 22c

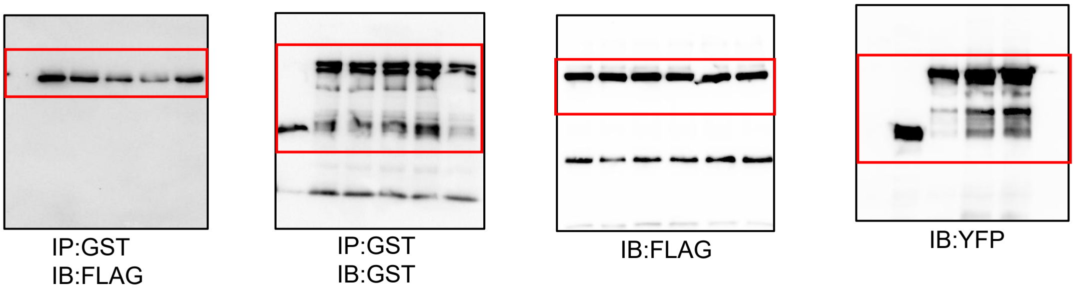

Supplementary Fig. 23b

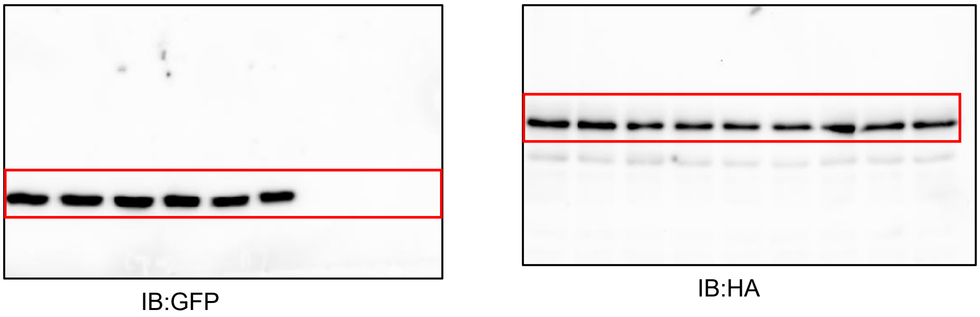

Supplementary Fig. 23b

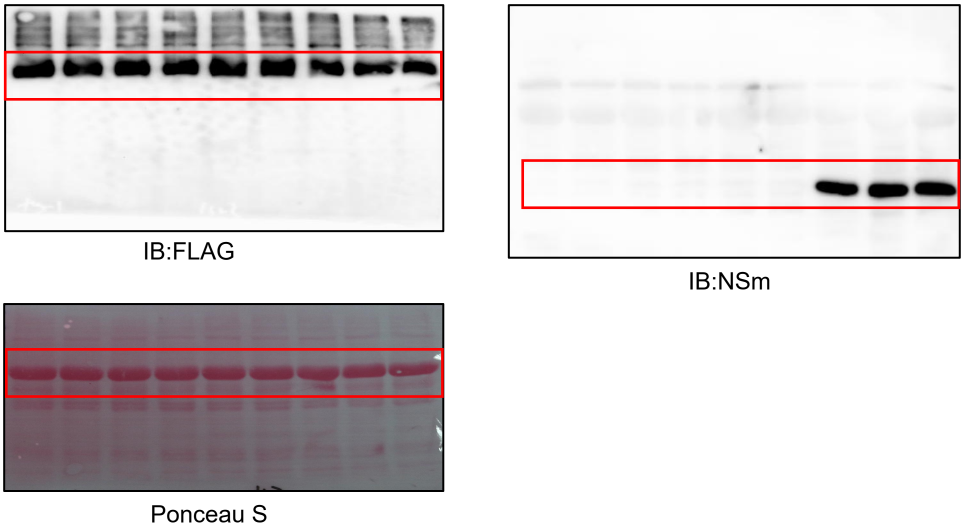

Supplementary Fig. 23d

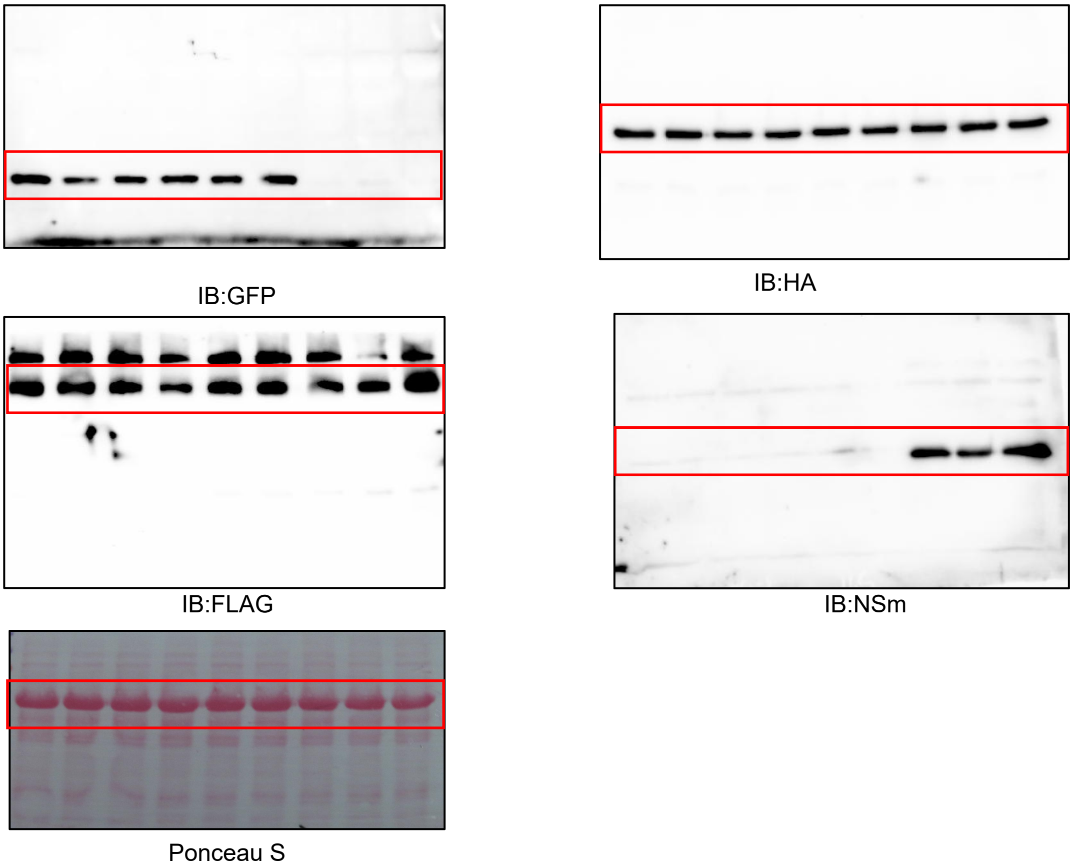

Supplementary Fig. 24b

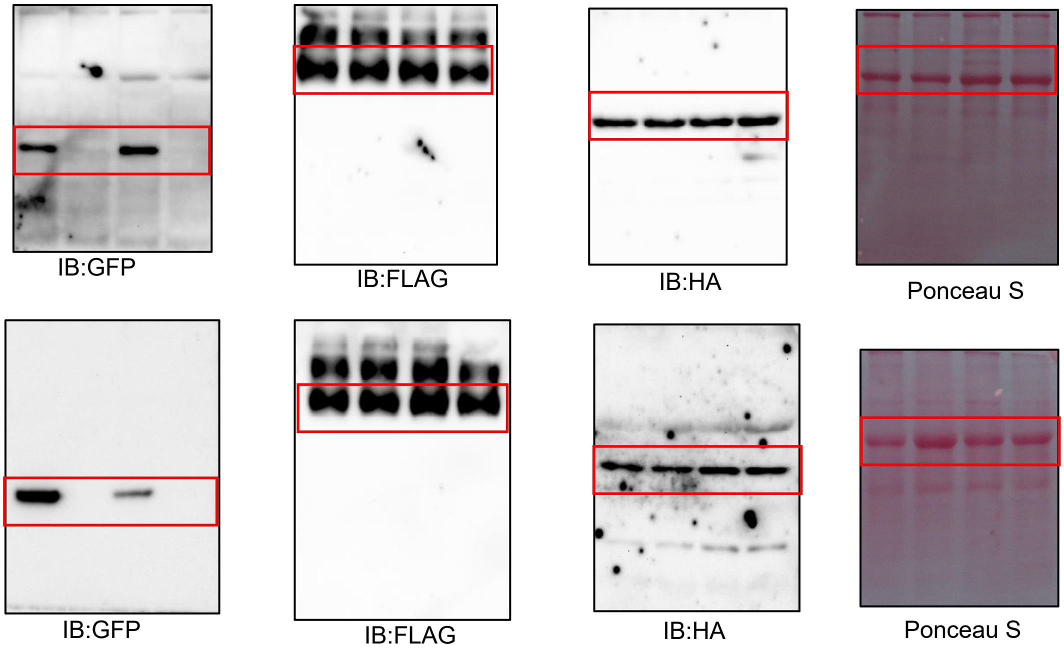

Supplement: Supplementary file 4 — Source Data [file 41467_2024_47364_MOESM4_ESM.zip › Source data/Original Blot Figures.pdf]
